# SIL1, the endoplasmic-reticulum-localized BiP co-chaperone, plays a crucial role in maintaining skeletal muscle proteostasis and physiology

**DOI:** 10.1242/dmm.033043

**Published:** 2018-05-10

**Authors:** Viraj P. Ichhaporia, Jieun Kim, Kanisha Kavdia, Peter Vogel, Linda Horner, Sharon Frase, Linda M. Hendershot

**Affiliations:** 1 Dept of Microbiology, Immunology, and Biochemistry, The University of Tennessee Health Science Center, Memphis, TN 38163, USA; 2 Dept of Tumor Cell Biology, St. Jude Children's Research Hospital, Memphis, TN 38105, USA; 3 Small Animal Imaging Center, St. Jude Children's Research Hospital, Memphis, TN 38105, USA; 4 Proteomics Facility, St. Jude Children's Research Hospital, Memphis, TN 38105, USA; 5 Dept of Pathology, St. Jude Children's Research Hospital, Memphis, TN 38105, USA; 6 Cell and Tissue Imaging Center, St. Jude Children's Research Hospital, Memphis, TN 38105, USA

**Keywords:** SIL1, Endoplasmic reticulum, Marinesco-Sjögren syndrome, Myopathy, Proteostasis collapse, PI3K-AKT-mTOR signaling

## Abstract

Mutations in *SIL1*, a cofactor for the endoplasmic reticulum (ER)-localized Hsp70 chaperone, BiP, cause Marinesco-Sjögren syndrome (MSS), an autosomal recessive disorder. Using a mouse model, we characterized molecular aspects of the progressive myopathy associated with MSS. Proteomic profiling of quadriceps at the onset of myopathy revealed that SIL1 deficiency affected multiple pathways critical to muscle physiology. We observed an increase in ER chaperones prior to the onset of muscle weakness, which was complemented by upregulation of multiple components of cellular protein degradation pathways. These responses were inadequate to maintain normal expression of secretory pathway proteins, including insulin and IGF-1 receptors. There was a paradoxical enhancement of downstream PI3K-AKT-mTOR signaling and glucose uptake in SIL1-disrupted skeletal muscles, all of which were insufficient to maintain skeletal muscle mass. Together, these data reveal a disruption in ER homeostasis upon SIL1 loss, which is countered by multiple compensatory responses that are ultimately unsuccessful, leading to *trans*-organellar proteostasis collapse and myopathy.

## Abstract (precis)

**Editor's choice:** This study provides molecular insights into the progressive myopathy and cellular compensatory responses attempted upon loss of SIL1, a component of the endoplasmic-reticulum-resident Hsp70 protein-folding machinery.

## INTRODUCTION

Nearly one-third of the human genome encodes proteins that enter the endoplasmic reticulum (ER) lumen, where they are often modified by N-linked glycans and fold into complex tertiary and quaternary structures that are stabilized by disulfide bonds (Braakman and Bulleid, 2011). This intricate maturation process is vulnerable to mistakes at any of these steps, which could result in aberrant proteins that are devoid of function at best, and prone to toxic aggregate formation at worst. The ER is populated with a host of molecular chaperones that bind directly to vulnerable unfolded proteins and serve to aid in their proper maturation by restricting off-pathway folding intermediates. Alternatively, if this process fails, some of these same chaperones can identify and target the unfolded or misfolded clients for degradation through a process referred to as ER-associated degradation (ERAD) (Ahner and Brodsky, 2004; Qi et al., 2017). These stringent ER quality control (ERQC) measures ensure that only properly modified, folded and assembled proteins are allowed to traffic to other organelles of the secretory pathway, to be expressed at the cell surface, or to be secreted (Ellgaard and Helenius, 2003; McCaffrey and Braakman, 2016). Intrinsic to the ERQC machinery is a feedback mechanism that allows the ER to respond to imbalances in normal protein folding by activating the unfolded protein response (UPR) in an effort to adjust and maintain ER homeostasis (reviewed in Walter and Ron, 2011).

ER chaperones can be grouped into two major families – the Hsp70 and the lectin chaperones – each having numerous cofactors that regulate their activity and interactions with nascent client proteins. Like all Hsp70 family members, the ER cognate BiP binds client proteins in an adenosine-nucleotide-regulated manner. The ATP-bound form of BiP readily engages clients, albeit with low affinity. This triggers ATP hydrolysis, transforming BiP into a high-affinity ADP-bound form (Wei et al., 1995). Two sets of cofactors further regulate BiP's ATPase cycle: ER-localized DnaJ-like proteins (ERDJs) and nucleotide exchange factors (NEFs). A subset of known ERDJs (ERDJ3-6) can directly bind clients, transfer them to BiP and assist in promoting either pro-folding or pro-degradation functions of BiP (Otero et al., 2010). Unfolded clients are protected from aggregation or misfolding while bound to BiP, but must be released in order to fold completely. This requires the action of NEFs that specifically release ADP from the BiP-client complex, allowing a new molecule of ATP to rebind. Consequently, the affinity of BiP for the bound client protein is lowered, leading to its release. Two ER-localized proteins have been identified that possess NEF activity: SIL1 (Chung et al., 2002) and GRP170 (Behnke et al., 2014). Both are ubiquitously expressed but do not appear to be completely redundant. While both SIL1 and GRP170 contribute to the release of cholera toxin from BiP, allowing it to be retrotranslocated to the cytosol (Williams et al., 2015), only GRP170 is able to stimulate the release of SV40 viral particles from BiP for their ER-to-cytosol transportation (Inoue and Tsai, 2015). Similarly, GRP170 has been reported to function in the release of two misfolded proteins from BiP (i.e. the NHK-variant of α1-antitrypsin and a transthyretin mutant), thereby promoting their degradation, whereas SIL1 does not appear to contribute in this case (Inoue and Tsai, 2016).

While unique functions for SIL1 in either BiP-mediated folding or degradation of endogenous clients have largely eluded identification, the discovery that mutations in *SIL1* are the leading cause of Marinesco-Sjögren syndrome (MSS) indicates that there are SIL1-specific functions (Anttonen et al., 2005; Senderek et al., 2005). MSS is an autosomal recessive, multisystem disorder that adversely affects Purkinje cells, leading to a profound cerebellar ataxia, and is further clinically characterized by progressive myopathy and bilateral cataracts, among other tissue pathologies (Ezgu et al., 2014; Horvers et al., 2013). Mutations in GRP170 have not yet been linked to any disease, but attempts to make a *GRP170*-null mouse were not successful, revealing it to encode an essential protein (Kitao et al., 2001). GRP170 is also a bona fide molecular chaperone (Behnke and Hendershot, 2014; Park et al., 2003). This might account for the mouse embryonic lethality observed upon its loss, because SIL1 has no known chaperone function that could compensate. However, it remains unclear why the lack of SIL1 causes no apparent problem in some tissues (Ichhaporia et al., 2014) while others are severely crippled, since both proteins are highly expressed in most secretory tissues (Chung et al., 2002; Weitzmann et al., 2007).

Our goal was to identify the molecular pathway(s) underlying the progressive myopathy associated with MSS. Two mouse models have been created in which the *Sil1* gene is disrupted in all tissues: one by a spontaneous transposon-mediated insertion known as the woozy mouse, *Sil1*^wz^, and the other by gene-trap methodology, referred to as the *Sil1*^Gt^ mouse model (Zhao et al., 2005). These mice have been reported to phenocopy at least some of the MSS-associated pathologies, including severe Purkinje cell loss leading to ataxia in both models (Zhao et al., 2005) and myopathy in the *Sil1*^wz^ mouse model (Roos et al., 2014). Using the *Sil1*^Gt^ mouse model, we observed a progressive loss of skeletal muscle mass and strength, which is characterized by a multifocal myogenic myopathy. Proteomic analyses revealed that the effects of SIL1 loss extended far beyond the ER, compromising all major organelles and pathways critical to normal muscle physiology. Consistent with a function for SIL1 in ERQC, we observed disruption of ER homeostasis, upregulation of ER chaperones and components of cellular protein degradation pathways, and aberrant expression of secretory pathway proteins that are critical for maintaining glucose and protein homeostasis in skeletal muscles. Our analyses provide molecular insights into why disruption of a component of the ERQC machinery results in loss of skeletal muscle mass, strength and function.

## RESULTS

### *Sil1*^Gt^ mice display evidence of a progressive myogenic myopathy

**Fig. 1.**
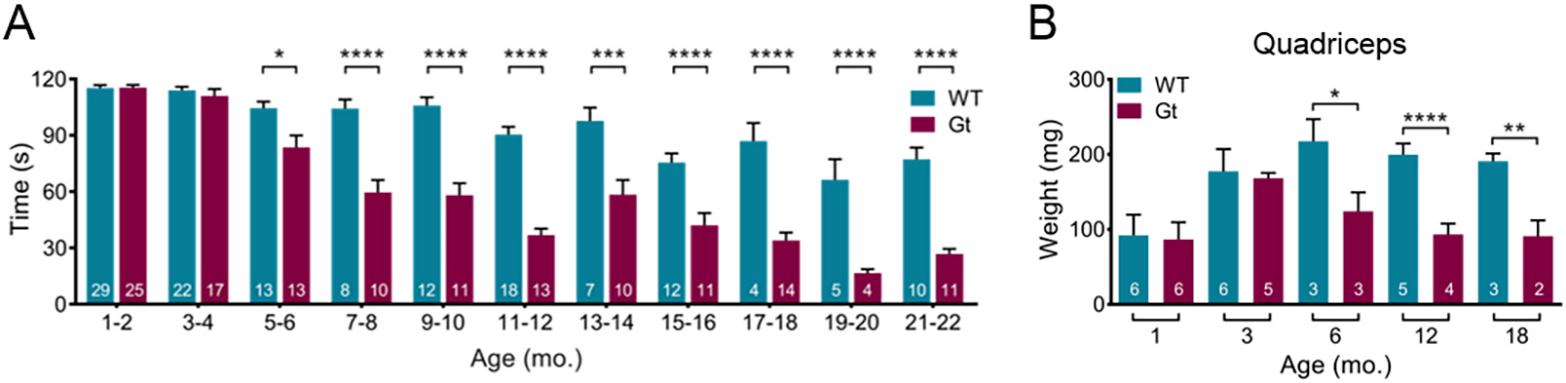
**SIL1 is required to maintain muscle strength and mass as mice age.** (A) Age-matched wild-type and *Sil1*^Gt^ mice ranging from 1 to 22 months were subjected to an inverted screen test for up to 120 s, and the duration of time they were able to hold onto the screen was measured. The number of mice in each group is indicated in white within the bars. Error bars indicate means±s.e.m. (B) Quadriceps femoris were obtained from wild-type and *Sil1*^Gt^ mice at the indicated ages. The number of mice included in each group is shown within the bars. Error bars indicate means±s.d. Statistical differences were computed using unpaired, two-tailed Mann–Whitney tests (A) or Student's *t*-tests (B), and are indicated as *P≤0.05, **P≤0.01, ***P≤0.001 and ****P≤0.0001.

As the *Sil1*^Gt^ mice aged, we noticed that they began to have difficulty holding on to the wired food tray while eating. We hypothesized that this might be indicative of a muscular weakness, similar to that observed in MSS patients (Sewry et al., 1988; Superneau et al., 1987) and in keeping with pathology and biochemical data obtained from *Sil1*^wz^ mice (Roos et al., 2014). We used an inverted screen test to examine limb muscle strength of *Sil1*^Gt^ mice and their wild-type littermates at ages ranging from 1 to 22 months (Fig. 1A). As early as 6 months, *Sil1*^Gt^ mice displayed a significantly reduced ability to hold on to the inverted screen, which became progressively worse with age. Necropsy revealed a corresponding decrease in *Sil1*^Gt^ quadriceps mass with age, which was not observed in soleus or heart (Fig. 1B and Fig. S1).

**Fig. 2.**
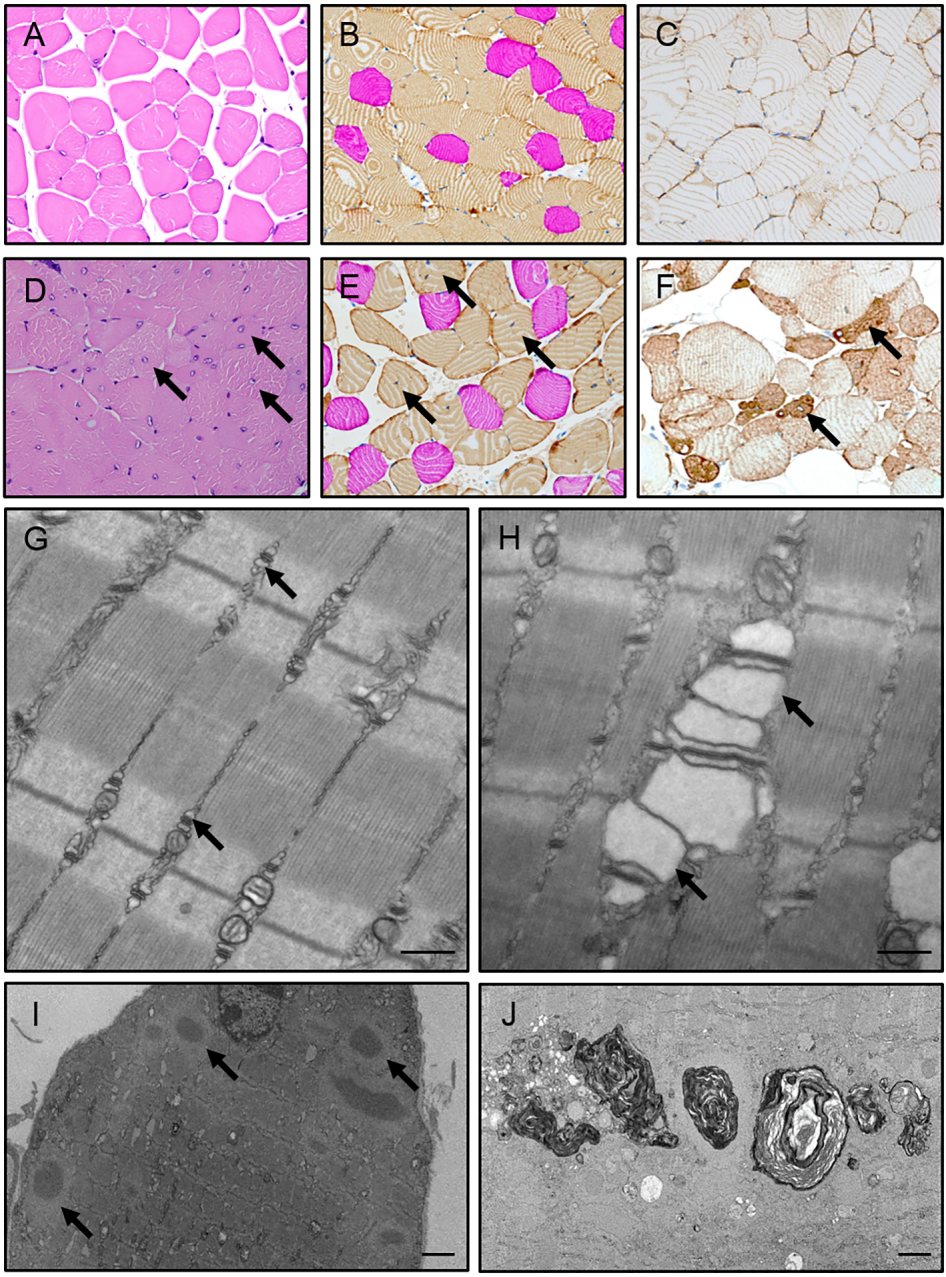
**The myopathy resulting from SIL1 loss predominantly affects fast myofibers.** Skeletal muscle cross-sections shown here were derived from 12-month wild-type (A-C) and *Sil1*^Gt^ (D-F) mice. (A,D) H&E staining revealed hypertrophic and atrophic fibers with internalized nuclei in the *Sil1*^Gt^ skeletal muscles (arrows), compared to peripheral nuclei in *Sil1*^WT^ samples. (B,E) Epaxial muscles were immunostained with fast (brown) and slow (pink) myofiber-specific MyHC antibodies. Arrows indicate myopathic myofibers with internalized nuclei, which stain positive for fast-myofiber-specific MyHC. (C,F) Desmin staining shows evidence of aggregation and loss of cyto-architecture in degenerating *Sil1*^Gt^ skeletal muscle fibers (arrows). (G,H) Representative electron micrographs from 10-month wild-type (G) and *Sil1*^Gt^ (H) bicep femoris displaying triads (arrows). (I,J) Electron micrographs from 10-month-old *Sil1*^Gt^ quadriceps demonstrating accumulation of cytoplasmic inclusion bodies (I) and membranous whorls (J) (indicated by arrows), all of which are characteristic of MSS. (A-F) Images were captured at the same 40× magnification. Scale bars: 400 nm (G,H); 2 µm (I,J).

To characterize the loss in muscle strength and mass, we conducted histological and electron microscopy (EM) analyses on a variety of muscle groups. Hematoxylin and eosin (H&E) staining showed that a subset of *Sil1*^Gt^ skeletal muscles had numerous fibers with internalized nuclei and myofibril disruption (Fig. 2A,D), and displayed evidence of adipose infiltration (Fig. S2C,D). Staining of skeletal muscles for laminin, a major component of the extracellular matrix, revealed muscular dystrophy in *Sil1*^Gt^ skeletal muscles, with a loss of the typical polygonal myofiber morphology and a wide range in myofiber diameter, including large, rounded hypertrophic fibers and shrunken, degenerating atrophic fibers (Fig. S2A,B). Immunostaining of skeletal muscle cross-sections for IgG (150 kDa) and IgM (900 kDa) did not show accumulation of these serum proteins in *Sil1*^Gt^ skeletal muscles, indicating that the hypertrophic myofibers maintained intact sarcolemmal integrity (Fig. S2E-H). Epaxial muscles are composed of both predominantly glycolytic fast myofibers and largely oxidative slow myofibers, which can be distinguished by the type of myosin heavy chain (MyHC) they express. Thus, MyHC immunostaining can be readily used to determine if there is any specificity in the type of myofibers that are affected upon SIL1 disruption. We found that the affected fibers were almost entirely limited to those expressing MyHC type II, indicative of fast-twitch glycolytic myofibers (Fig. 2B,E). In keeping with this observation, the soleus, which is predominantly composed of oxidative myofibers, did not appear to be affected in any of the analyses performed, whereas the biceps brachii, gastrocnemius, quadriceps femoris and tibialis anterior, which are all largely made up of glycolytic myofibers, showed significant pathological changes (as observed in Fig. 2 and Fig. S2). The heart, composed of specialized oxidative cardiomyocytes, also remained unaffected in these mice, arguing that glycolytic myofibers are uniquely vulnerable to SIL1 loss. Immunostaining for desmin, an intermediate filament protein, revealed significant disruption of myofiber architecture and accumulation of this protein in degenerating *Sil1*^Gt^ myofibers (Fig. 2C,F). EM analyses were consistent with these findings, and additionally revealed dramatically dilated sarcoplasmic-reticular-triads in atrophying fibers (Fig. 2G,H), degenerating nuclei, rare mitochondrial abnormalities and a double membranous structure enveloping the nucleus (Fig. S2K-M). We observed electron-dense material and cytoplasmic bodies in various myofibers, suggestive of protein aggregation, and an accumulation of membranous whorls, indicative of autophagosomes (Fig. 2I,J). Together, these alterations strongly resemble ultrastructural changes observed in muscle biopsies of individuals with MSS (Goto et al., 1990; Sewry et al., 1988) and quadriceps from *Sil1*^wz^ mice (Roos et al., 2014), and explain the decreased muscle mass and strength occurring in *Sil1*^Gt^ mice. The dispersed, multifocal and dystrophic myopathy we observed in *Sil1*^Gt^ glycolytic skeletal muscles is most compatible with a myogenic myopathy. This is in contrast to the clustered myopathy with angular cross-sectional myofiber morphology reported in *Sil1*^wz^ mice, which is more indicative of a neurogenic origin (Filézac de L'Etang et al., 2015). Furthermore, H&E-stained lumbo-sacral spinal sections did not reveal any obvious neurogenic pathology (Fig. S2I,J), arguing against a major contribution of motor neuron defects in the *Sil1*^Gt^ progressive myopathy and revealing some differences in these two genetic models.

### SIL1 disruption leads to widespread changes in the quadriceps proteome

**Fig. 3.**
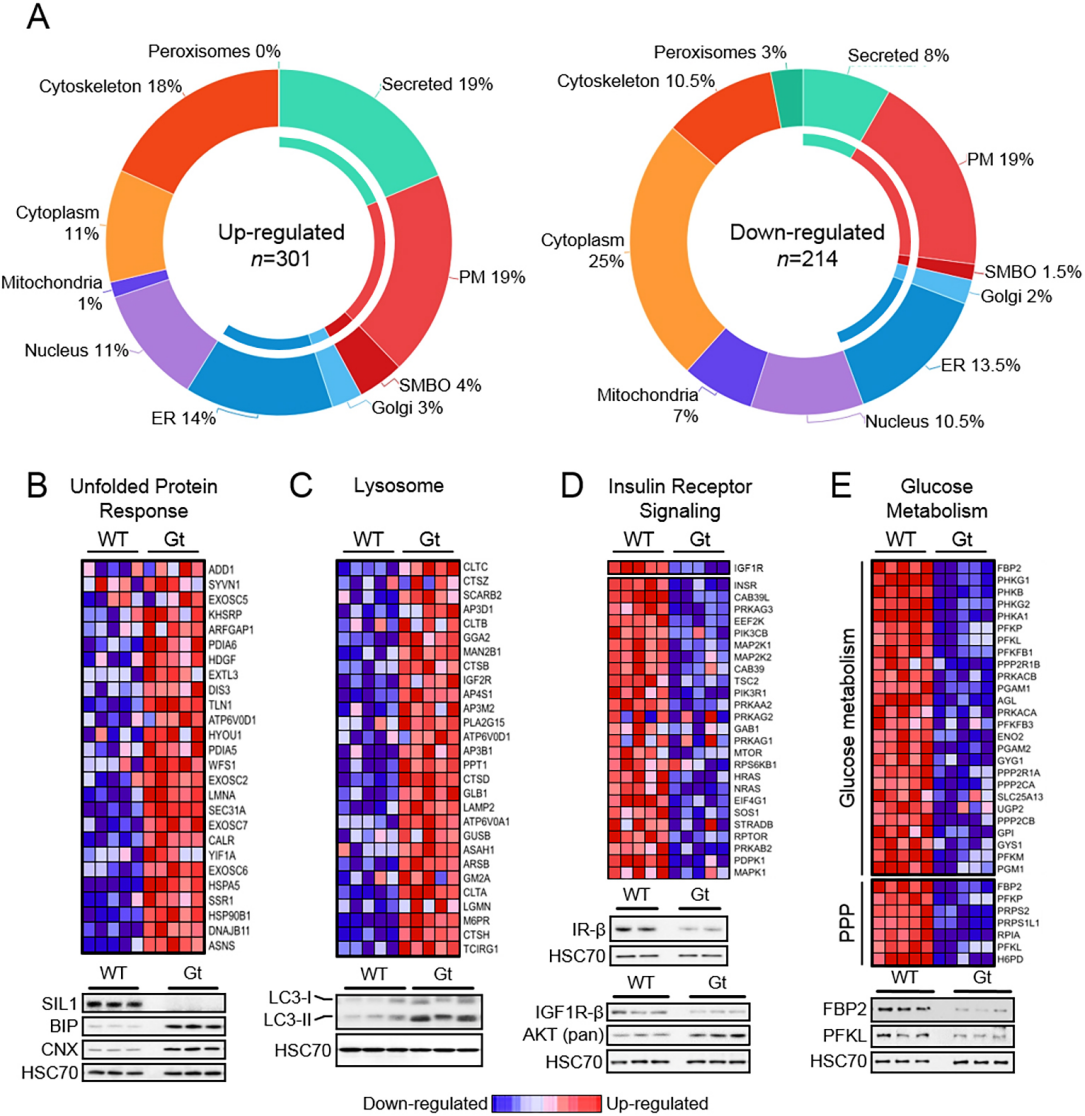
**SIL1 disruption perturbs all major cellular organelles and pathways critical for normal quadriceps function.** (A) Graphical representation of the organellar distribution of proteins that were identified by proteomic analyses to be significantly (log_2_ ratio <−0.5 or >0.5; *P*<0.01) upregulated (left) or downregulated (right) in quadriceps from 6-month-old Sil1_Gt_ mice relative to that from wild-type mice (*n*=5). Inner band indicates compartments of the secretory pathway. SMBO, single membrane bound organelles; PM, plasma membrane. (B-E) Heat maps of GSEA-identified gene-sets that are enriched (B,C) or depleted (D,E) in the *Sil1*^Gt^ quadriceps. PPP, pentose phosphate pathway. Subsets of proteins for each gene-set were selected for verification by western blotting and are shown below the heat maps.

In an unbiased approach to understand the *Sil1*^Gt^-associated myopathy at the molecular level, we performed proteomic analyses on quadriceps derived from wild-type (*Sil1*^WT^) and *Sil1*^Gt^ male mice at 6 months of age. This was the age at which the myopathy first became physiologically and pathologically evident. Among skeletal muscles strongly affected by SIL1 disruption, quadriceps femoris were our tissue of choice due to their composition of predominantly glycolytic myofibers, the availability of sufficient amounts of protein due to their relatively large mass, and the ease of dissection. Five mice of each genotype were sampled. A total of 5512 proteins were identified, of which 5384 could be quantified. Bioinformatics analyses identified 515 proteins whose expression was significantly altered (log_2_ ratio <−0.5 or >0.5; *P*<0.01) in *Sil1*^Gt^ quadriceps, relative to those from wild-type mice. Of these, 301 proteins were upregulated and 214 were decreased in expression (Table S2). Manual curation of these proteins, sorted by their resident subcellular compartments according to the GeneCards database (http://www.genecards.org/) (Stelzer et al., 2016), revealed that loss of the ER co-chaperone SIL1 led to changes in protein expression extending to all major cellular compartments (Fig. 3A). Gene-set enrichment analysis (GSEA) identified a significant number of proteins, belonging to signaling pathways and functions critical to muscle physiology, including insulin receptor (IR) signaling, glucose metabolism and cation channel activity, to be altered in the quadriceps of 6-month-old *Sil1*^Gt^ mice. We verified the indicated changes in expression of a number of these proteins by western blotting (Fig. 3B-E and Fig. S3). Altered expression of ER chaperones, secretory pathway proteins that are synthesized in the ER, and elements of the cellular degradation machinery could be readily anticipated by loss of a component of the ER protein folding machinery. However, possible mechanisms leading to altered expression of proteins throughout other organelles was not immediately clear.

### Loss of SIL1 disrupts ER homeostasis as mice age

**Fig. 4.**
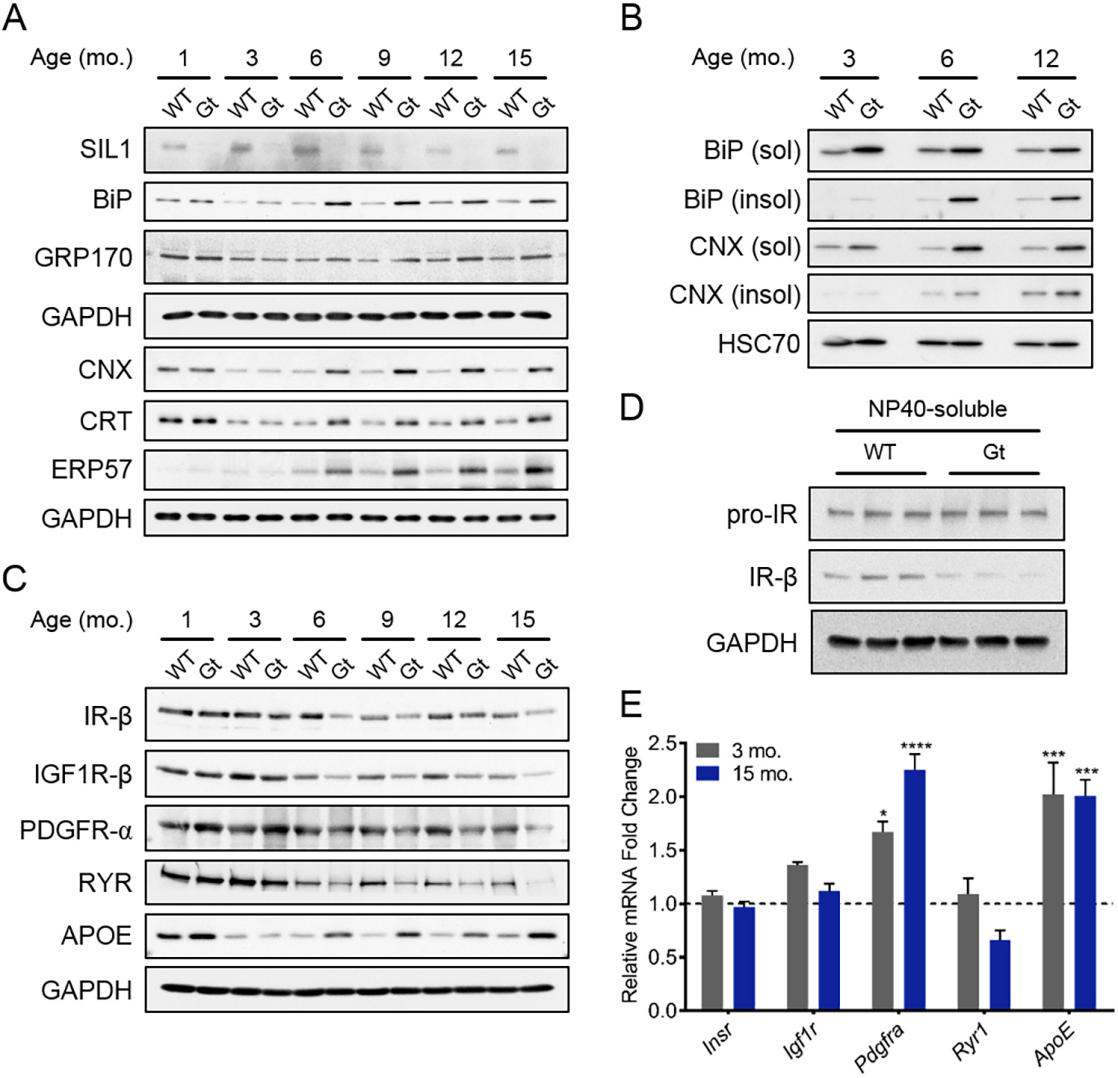
**SIL1 is required to maintain ER proteostasis in quadriceps as mice age.** (A) Wild-type and *Sil1*^Gt^ quadriceps lysates (*n*=3-5 pooled), solubilized in 8 M urea, isolated from mice at the depicted ages were normalized for an equivalent amount of total protein, pooled and subjected to reducing SDS electrophoresis, followed by transfer to PVDF membranes, and blotting with the indicated immune reagents for ER chaperones and co-chaperones. GAPDH serves as a control for loading. (B) Wild-type and *Sil1*^Gt^ quadriceps obtained from mice at the designated ages were lysed with NP40 lysis buffer. The resulting NP40-soluble and NP40-insoluble fractions were processed for western blotting with BiP and calnexin antisera. HSC70 serves as a loading control for the NP40-soluble fraction. (C) Urea-solubilized quadriceps lysates derived from mice of the indicated genotypes and ages were probed with antibodies to proteins that traffic through the secretory pathway. (D) NP40-solubilized quadriceps lysates from 12-month-old mice were processed for western blotting to detect the insulin receptor precursor (pro-IR) and the mature subunit (IR-β). (E) Graphical representation of normalized mRNA fold changes of proteins probed in C from *Sil1*^Gt^ quadriceps, relative to wild-type quadriceps (*n*=3) (set to 1; indicated by the dotted line) at 3 (gray) and 15 (blue) months. Error bars indicate means±s.e.m. Statistical differences were computed using unpaired, two-tailed Student's *t*-tests, and are indicated as *P≤0.05, ***P≤0.001 and ****P≤0.0001.

To begin to understand the widespread changes in protein expression that occurred upon SIL1 disruption, we examined time points both prior to and after the onset of muscular weakness and began with proteins that contribute to ERQC, since this is where SIL1 functions. We combined quadriceps lysates from at least three male mice, for each age shown, to obtain an average expression for the proteins queried. The folding of secretory pathway proteins entering the ER as nascent chains is both aided and monitored by a host of molecular chaperones and their cofactors. Beginning as early as 3 months, we observed a slight increase in BiP expression in *Sil1*^Gt^ quadriceps, which became more dramatic by 6 months and remained elevated (Fig. 4A and Fig. S4C). GRP170, the other ER NEF (Weitzmann et al., 2006), showed a modest increase in expression beginning at 6 months, whereas the lectin chaperones (calnexin and calreticulin) and their cofactor, ERP57, were more robustly upregulated. The delayed upregulation of these proteins suggested that loss of SIL1 led to a progressive disruption of ER homeostasis that was initially dealt with. These proteins can be upregulated at the transcriptional level by activation of the UPR due to the accumulation of unfolded proteins in the ER. We found evidence for the upregulation of targets of all three upstream transducers (PERK, ATF6 and IRE1) in *Sil1*^Gt^ muscles relative to age-matched wild-type mice (Fig. S4A-C). As an independent means of detecting disruptions in ER homeostasis, we examined the relative solubility of BiP and calnexin. Both are normally soluble ER chaperones that bind unfolded proteins to help maintain them in a soluble form. However, if they are unable to do so, these chaperones can become trapped in detergent-insoluble complexes with client proteins. Unlike quadriceps, which were lysed in 8 M urea for proteomic and kinetics-of-onset studies, we used milder, NP40-mediated lysis conditions and examined NP40-soluble and -insoluble fractions at three time points. As early as 3 months, a very small amount of BiP was detected in the NP40-insoluble fraction of *Sil1*^Gt^ quadriceps, which became more prominent in the 6 and 12 months samples (Fig. 4B). It is noteworthy that, as the *Sil1*^WT^ mice aged, a small amount of BiP was also detected in the insoluble fraction. This is in agreement with reports of age-related disruptions in protein homeostasis, also referred to as proteostasis, which is a hallmark of skeletal muscle aging (Demontis et al., 2013; López-Otín et al., 2013).

Consistent with the observation of dilated sarcoplasmic-reticular- triads, the presence of BiP in the insoluble fraction implied that loss of SIL1 was likely to affect the maturation of cargo proteins that are synthesized in the ER and traffic through the secretory pathway. Indeed, the proteomics data indicated that secreted (19% upregulated, 8% downregulated) and plasma membrane (19% upregulated, 19% downregulated) proteins represented a significant fraction of proteins with altered expression (Fig. 3A). We chose to investigate four integral membrane proteins that showed reduced expression, including three subunits of heterodimeric cell surface receptors – insulin receptor β chain (IR-β), insulin-like growth factor 1 receptor β chain (IGF1R-β) and the PDGF receptor α chain (PDGFR-α) – as well as ryanodine receptors (RYRs), which are transmembrane proteins that form calcium channels in the sarcoplasmic reticulum. Examination of quadriceps lysates at various time points confirmed a reduction in the expression of all four membrane proteins, although the kinetics and magnitude of these changes varied slightly (Fig. 4C and Fig. S4D). We quantified their mRNA levels to ensure that these changes were not due to a transcriptional effect. IR-β and IGF1R-β transcripts were not significantly altered at either time point tested in *Sil1*^Gt^ mice, whereas PDGFR-α transcripts were actually increased compared to that in *Sil1*^WT^ skeletal muscles (Fig. 4E). In fact, when NP40-soluble protein lysates from 12-month quadriceps were analyzed, although the IR precursor levels were nearly identical to those in wild-type muscles, we observed a significant reduction in mature IR-β subunit expression in *Sil1*^Gt^ muscles (Fig. 4D and Fig. S4F). In the case of IGF1R, both the precursor and mature forms were decreased (Fig. S4E,F). Thus, the reduced expression of these three proteins was likely a result of decreased protein stability due to disrupted ER homeostasis. However, *Ryr1* mRNA was modestly reduced, making it more difficult to attribute the extent of change in protein levels to a folding defect. Conversely, our proteomic data indicated that the levels of multiple apolipoproteins (APOM, APOA1, APOA2, APOD and APOE), which are secreted proteins (Hartwig et al., 2014), were increased in *Sil1*^Gt^ quadriceps. Indeed, western blotting for cellular APOE, which has been found to abnormally accumulate in inclusion body myositis (Mirabella et al., 1996), showed a readily detectable increase as early as 6 months and remained elevated throughout the later time points (Fig. 4C and Fig. S4D). While *ApoE* transcripts were upregulated in *Sil1*^Gt^ samples, they did not change between 3 and 15 months of age, whereas the protein levels increased significantly at 15 months compared to 3 months (Fig. 4E), implying differences in post-translation regulation of this protein in *Sil1*^Gt^ myofibers. Importantly, unlike membrane proteins, which remain cell-associated when properly folded, secretory proteins exit the cell. Thus, a faster rate of transport for the properly folded APOE protein in the *Sil1*^WT^ muscle compared to the rate at which a misfolded protein is recognized and targeted for degradation could account for the increased APOE protein levels in *Sil1*^Gt^ skeletal muscles.

### Evidence for inadequate compensation by cellular degradation systems

**Fig. 5.**
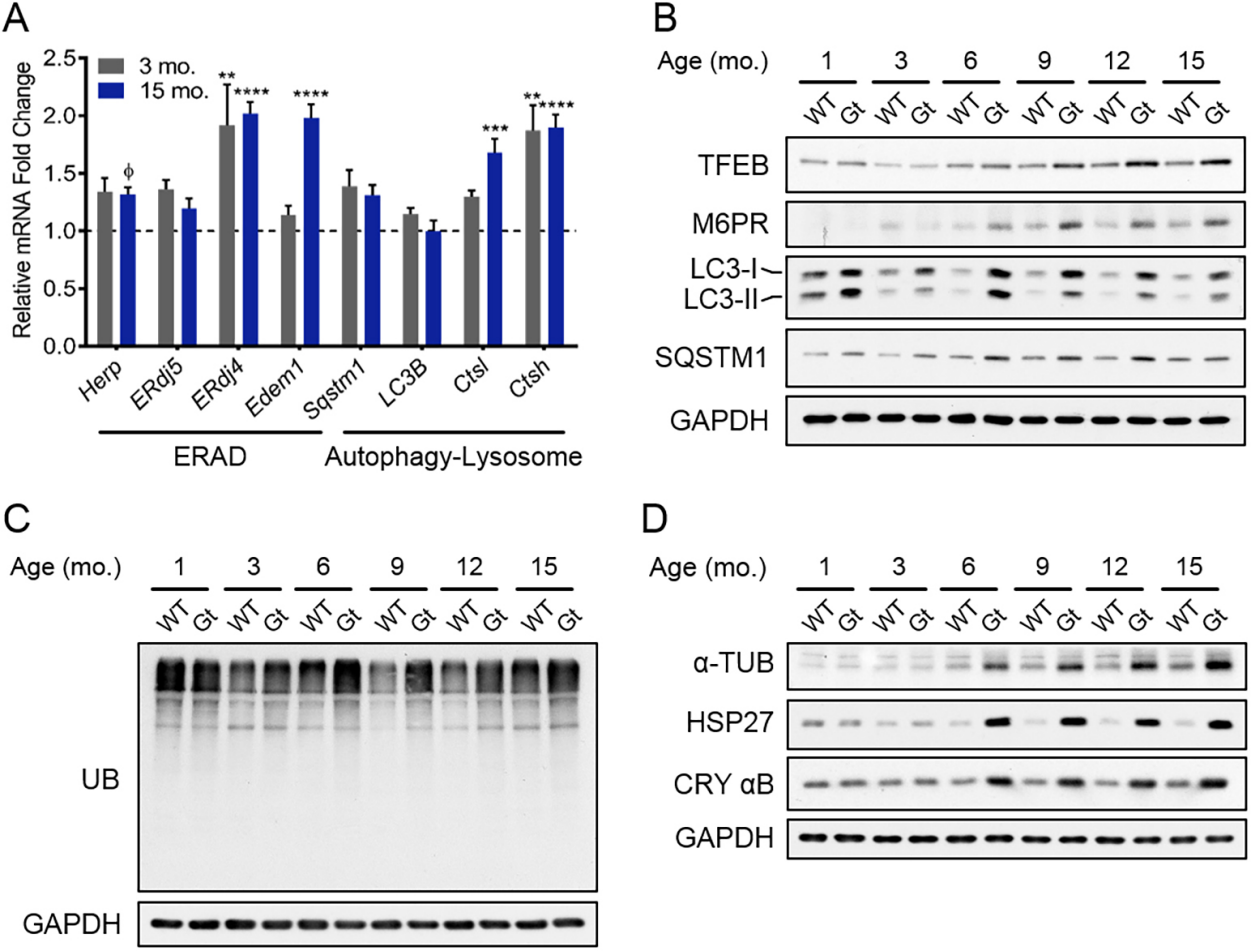
**Loss of SIL1 results in compensating increases in all cellular protein degradation systems.** (A) Graphical representation of normalized mRNA fold changes of ERAD components (*Herp*, *ERdj5*, *ERdj4* and *Edem1*), crucial mediators of autophagy (*Sqstm1*/*p62* and *LC3B*) and lysosomal hydrolases [cathepsin L (*Ctsl*) and cathepsin H (*Ctsh*)] in quadriceps (*n*=3) obtained at 3 (gray) and 15 (blue) months of age. Error bars indicate means±s.e.m. Statistical differences were computed using unpaired, two-tailed Student's *t*-tests, and are indicated as ^ɸ^*P*<0.1 but >0.05, **P≤0.01, ***P≤0.001 and ****P≤0.0001. (B,C) Urea-lysed, pooled cellular extracts (*n*=3-5) isolated at the indicated ages were probed for markers of autophagy and lysosomal biogenesis (B), and also for the accumulation of ubiquitinated proteins (C) by western blotting. GAPDH is a control for equivalent loading in both panels. (D) Lysates were probed for the indicated proteins involved in sequestering misfolded proteins and maintaining the cytoskeleton.

Based on the demonstration of altered expression of several secretory pathway proteins, and because BiP and the lectin chaperones participate in both the folding of clients and the targeting of misfolded proteins for degradation, we queried the expression of their ERAD-associated cofactors, ERDJ4, ERDJ5 and EDEM1, respectively. *ERdj4* and *Edem1* transcripts were significantly increased in *Sil1*^Gt^ quadriceps when compared to age-matched *Sil1*^WT^ tissue, particularly at the later time point (Fig. 5A). Transcripts for *Herp/Herpud1*, a component of the retrotranslocon, displayed a slight increase, which was not statistically significant, as did HERP protein levels (data not shown). Together, these data indicate an increased demand for the disposal of misfolded ER proteins. Improperly matured or aggregated ER proteins, as well as damaged ER, can also be disposed of by lysosomal and autophagy pathways. Consistent with a role for these systems in managing ER proteostasis failures, we detected a relative increase in transcription factor EB (TFEB), a key mediator of lysosomal biogenesis, as early as 6 months (Fig. 5B and Fig. S5K). Consistent with this, we detected multiple TFEB targets that play a role in lysosomal function to be significantly upregulated by our proteomics study (Fig. S5A and Table S3). We validated one of these, the mannose-6-phosphate receptor (Fig. 5B and Fig. S5K), which transports lysosomal enzymes and clients to lysosomes (Sardiello et al., 2009). Transcripts for cathepsin L and cathepsin H, two lysosomal hydrolases, were also significantly increased (Fig. 5A). TFEB overexpression has also been reported to drive mitochondrial biogenesis (Mansueto et al., 2017). Although our proteomics data captured 46 TFEB-regulated proteins involved in this process, most were either unchanged or diminished (Fig. S5B), arguing that TFEB did not apparently reach sufficiently high levels to contribute to their expression. Proteins and damaged organelles are also delivered to the lysosome via autophagy. Both the unmodified and lipidated forms of the microtubule-associated protein 1 light chain 3 beta (LC3B), the latter of which initiates the formation of autophagic vesicles, were significantly upregulated in *Sil1*^Gt^ quadriceps at even the earliest time point queried. Similarly, we observed a modest increase in the levels of the autophagic adaptor protein p62 (also known as SQSTM1), which promotes the sequestration of ubiquitinated target proteins into autophagosomes, leading to lysosomal degradation of both p62 and associated proteins (Fig. 5B and Fig. S5K). This increase in the protein levels of LC3B and p62 was not due to a corresponding increase in their transcripts (Fig. 5A). In addition, we detected the presence of LC3B- and p62-positive puncta in *Sil1*^Gt^ skeletal muscles by immunohistochemistry (IHC) (Fig. S5C-H). Thus, the accumulation of unmodified LC3B-I and p62 by western blotting, cellular aggregation of these two proteins in puncta, and build-up of autophagosomes observed by EM together indicate impaired autophagy, which precedes the stimulus for lysosomal biogenesis in *Sil1*^Gt^ skeletal muscles. Correspondingly, we observed a striking increase in *Fgf21* mRNA (Fig. S6H), a myokine that has been implicated in metabolic adaptations to autophagy inhibition and ER stress (Kim and Lee, 2015).

Although our data show an upregulation of components involved in cellular clearance pathways in response to SIL1 disruption, this was apparently not sufficient to dispose of all misfolded proteins, as *Sil1*^Gt^ quadriceps samples also displayed a greater accumulation of ubiquitinated proteins relative to *Sil1*^WT^ samples, beginning at 3 months of age and extending throughout the time course measured (Fig. 5C and Fig. S5L). This taxation of the ubiquitin-proteasome system likely also compromises the turnover of proteins in other organelles, including the cytosol, as indicated by the upregulation of two cytosolic small heat shock proteins (sHSP), HSP27 and αB-crystallin (Fig. 5D and Fig. S5L), coinciding with the accumulation of ubiquitinated proteins in the *Sil1*^Gt^ samples. In addition to their primary roles in chaperoning multiple cytoskeletal proteins, these sHSPs play a more general cytoprotective role by sequestering cytosolic protein aggregates (Dubińska-Magiera et al., 2014), which is in keeping with the presence of these sHSP chaperones in the NP40-insoluble fraction (Fig. S5I,J) in *Sil1*^Gt^ quadriceps. We also observed an increase in the cytoskeletal proteins α-tubulin (Fig. 5D) and desmin (Figs. S3B and S5G,H), which rely on sHSPs to fold correctly (Arrigo et al., 2007). If the protein-folding capacity of these sHSPs becomes limiting, it is likely to impinge on client folding, which is consistent with desmin aggregation in *Sil1*^Gt^ skeletal muscles (Fig. 2F).

### Disruption of SIL1 has profound effects on components of the insulin signaling pathway

**Fig. 6.**
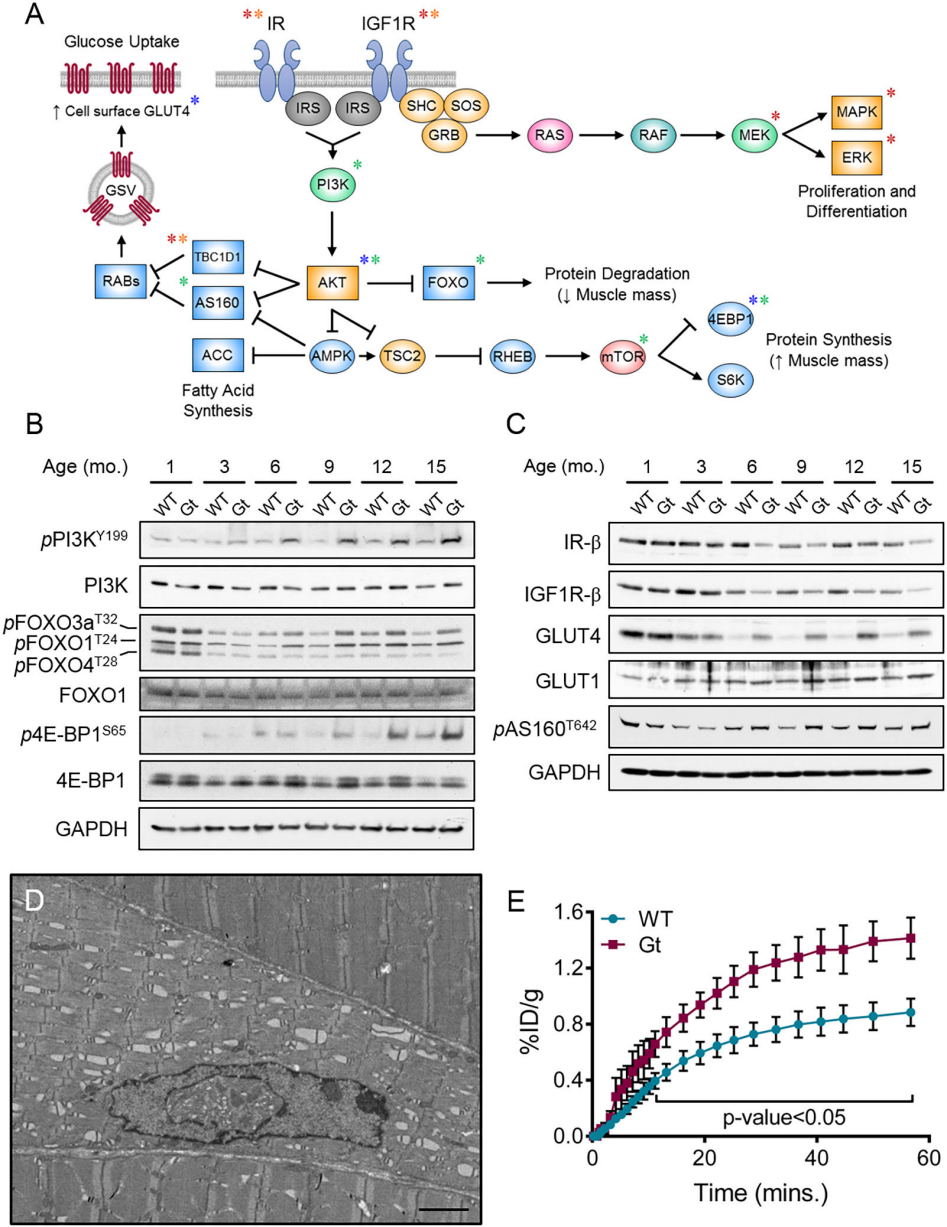
**Loss of SIL1 has profound effects on IR signaling.** (A) Schematic of the IR/IGF1R signaling pathway. Asterisks indicate proteins identified to be decreased by proteomics (red *), western-blotting-validated decreases (orange *) and increases (blue *), and proteins displaying increased basal phosphorylation (green *), relative to *Sil1*^WT^ levels. (B,C) Pooled, urea-solubilized quadriceps lysates derived from mice at the depicted ages (*n*=3-5) were analyzed by western blotting with antibodies against multiple components of the IR- and IGF1R-signaling pathways. GAPDH serves as a control for equivalent loading. (D) Representative gastrocnemius electron micrograph from 10-month-old *Sil1*^Gt^ mice displaying myopathic alterations very similar to those observed in the quadriceps, including degenerating nuclei and dilated triads. Scale bar: 2 µm. (E) Graphical representation of ^18^F-FDG uptake over 60 min in wild-type (blue) and *Sil1*^Gt^ (red) gastrocnemii (*n*=4) assayed by PET-µCT. Statistical differences were computed using unpaired, two-tailed Student's *t*-tests, and are indicated in the graph. %ID/g, % injected dose per gram body weight.

Insulin and IGF-1 signaling in skeletal muscles help regulate systemic glucose homeostasis and maintain muscle mass (reviewed in Ramalingam et al., 2013 and Schiaffino et al., 2013). These hormones signal through two highly homologous tyrosine kinase receptors that activate the PI3K-AKT pathway (Fig. 6A). We had verified that both IR-β and IGF1R-β expression were decreased in the *Sil1*^Gt^ quadriceps beginning at 6 months (Fig. 4C). Our proteomic data suggested that this decrease was accompanied by changes in the expression of numerous downstream components of the IR/IGF1R signaling pathway, including an unexpected increase in the levels of total AKT (Fig. 3D). We also observed reduced levels of key regulatory enzymes of glucose metabolism, including those involved in gluconeogenesis, glyconeogenesis, glycolysis and the pentose phosphate pathway (Fig. 3E). Thus, we examined the IR/IGF1R signaling pathway more closely in quadriceps from *ad libitum*-fed mice, which were the source of all our studies to this point. Paradoxically, we observed increased phosphorylation of their downstream target, PI3K, on tyrosine 199 beginning at 6 months and continuing through later time points (Fig. 6B and Fig. S6C,D). To better understand the significance of PI3K activation, we focused on the phosphorylation status of a number of both direct and indirect effectors of this kinase (Gingras et al., 2004). First, due to their respective contribution to skeletal muscle atrophy and translation repression, the FOXO transcription factors and 4E-BP1 were examined. A relative increase in phosphorylation was observed for FOXO1, FOXO3a and 4E-BP1 on the AKT- and mTOR-dependent residues, respectively, which in each case suppresses their activity. Reduced transcriptional activity of FOXO is further supported by decreased levels of two of its targets, the E3 ubiquitin ligases *Atrogin-1* and *MuRF1*, at the latter time point (Fig. S6G). Together, these data demonstrate that the aberrant increase in PI3K activation in the *Sil1*^Gt^ skeletal muscle is driving downstream responses, which are likely to limit the loss of muscle strength and mass.

Despite a sustained increase in PI3K-AKT signaling, the *Sil1*^Gt^ mice continued to lose muscle mass and strength as they aged. Although we did not observe evidence of an overt mitochondrial phenotype in these mice, impairment in energy production can lead to myopathy. Thus, the pronounced activation of AMPK, an energy stress sensor, can inhibit mTOR-mediated signaling, leading to a decrease in skeletal muscle mass. We checked the activation status of AMPK and its downstream target, acetyl-CoA carboxylase (ACC). Although there was a modest increase in AMPK phosphorylation at two time points, this did not correspond to a significant increase in phosphorylation of its target, ACC (Fig. S6B,E), suggesting that decreased energy production was not a significant driver of this myopathy. We also examined transcripts of GADD45A, a critical mediator of skeletal muscle atrophy, because it can be upregulated by the PERK pathway (Ebert et al., 2012), which is in keeping with our observation that CHOP, another PERK target, was induced (Fig. S4B). Indeed, there was a significant increase in *Gadd45a* mRNA in *Sil1*^Gt^ quadriceps as early as 3 months, which continued to climb as these mice aged, as well as a modest increase in *Ncam1*, a marker of atrophy (Fig. S6G). Two other mediators of atrophy, *Atrogin-1* and *MuRF1*, were not induced, likely due to the suppression of FOXO by AKT, thus resulting in a dampened atrophic response. This, coupled with the absence of a regeneration response driven by an increase in PAX7, MYOD1 and myogenin (Fig. S6G), apparently cause the slow, progressive myopathy observed in *Sil1*^Gt^ mice.

We also observed a dramatic increase in the levels of GLUT4, the insulin-responsive glucose transporter, beginning at 6 months, whereas GLUT1, which is an insulin-independent glucose transporter, was only modestly increased (Fig. 6C and Fig. S6F). The small increase in GLUT1 expression may be due to a similar increase in its transcripts, whereas *Glut4* mRNA levels were actually slightly decreased (Fig. S6H), suggesting post-translational stabilization of this transporter. A significant pool of GLUT4 is maintained in intracellular vesicles that can be rapidly deployed to the cell surface to allow glucose uptake in response to insulin signaling through the IR (Fig. 6A). This relocation of GLUT4 is negatively regulated by two GTPase-activating proteins, TBC1D1 and AS160 (TBC1D4), and they are in turn inactivated by phosphorylation through the insulin signaling pathway (reviewed in Rowland et al., 2011). We observed a slight steady-state increase in phospho-AS160 beginning at 6 months (Fig. 6C) and a significant decrease in *Tbc1d1* transcripts in our 15-month samples (Fig. S6H). Together, this indicates a reduction in both negative regulators of GLUT4 transport, arguing for a plausible increase in the cell surface localization of GLUT4 in *Sil1*^Gt^ quadriceps.

To understand the physiological relevance of increased GLUT4 levels and an inhibition or reduction of the negative regulators of GLUT4 trafficking under steady-state conditions, we measured basal glucose uptake rate in skeletal muscles using ^18^F-labeled fluorodeoxyglucose (^18^F-FDG) PET-μCT. Due to technical limitations, we were unable to analyze the signal originating from quadriceps, our tissue of choice. Thus, as an alternative, we monitored ^18^F-FDG uptake in gastrocnemii, which are also primarily glycolytic skeletal muscles and display a pathology similar to that observed in quadriceps upon SIL1 disruption (Fig. 6D). We observed an accelerated rate and increased magnitude of glucose uptake in gastrocnemii of *Sil1*^Gt^ mice, compared to age-matched wild-type mice (Fig. 6E). This indicates that the observed increases in basal phosphorylation of multiple components of the AKT pathway indeed represent functional activation of this signaling cascade and likely provides at least some compensation in the face of decreased IR-β and IGF1R-β protein expression.

## DISCUSSION

**Fig. 7.**
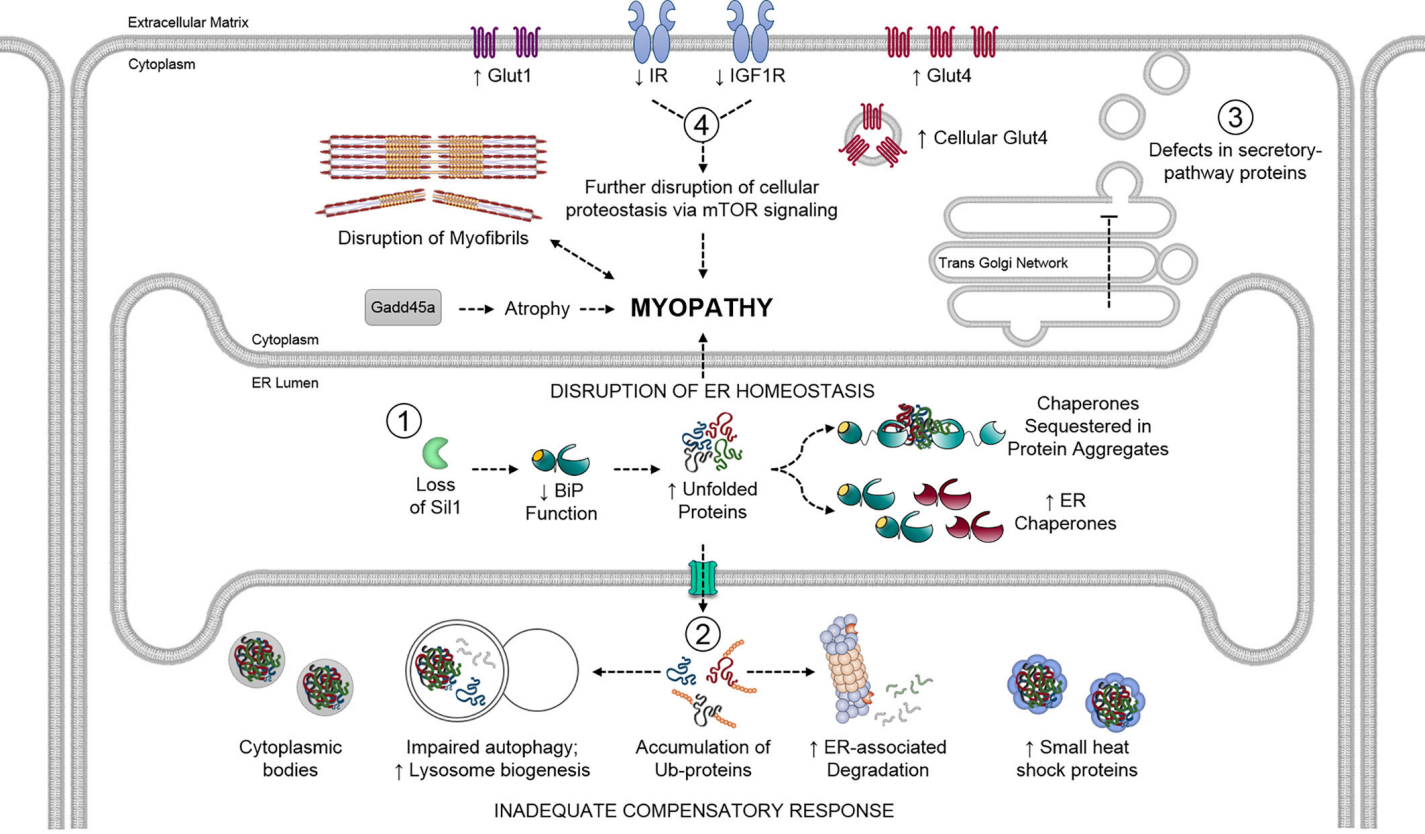
**Schematic model describing the molecular responses to SIL1 loss in skeletal muscles leading to myopathy.** (1) A functional depletion of SIL1 compromises the chaperoning ability of BiP, leading to an increase in chaperones in an attempt to maintain normal protein folding in the ER. However, this compensation is insufficient, resulting in client misfolding and aggregation. (2) Degradation pathways are upregulated to deal with accumulating misfolded proteins but are heavily taxed, leading to the build-up of ubiquitinated proteins and induction of small heat shock proteins to sequester aggregates. (3) In keeping with SIL1's role in protein folding, disruption of ER homeostasis adversely affects maturation of secretory pathway clients in the ER. (4) Consequentially, this results in a decrease in the amount of some clients, such as IR-β and IGF1R-β, which play critical roles in maintaining skeletal muscle protein and glucose homeostasis. Disruption of normal signaling via these axes, in combination with other factors, such as an increase in GADD45A levels, eventually shifts the balance between anabolic and catabolic cellular mechanisms towards catabolic, atrophy-causing pathway(s) that lead to cyto-architecture disruption as well as decreased muscle mass and strength in the *Sil1*^Gt^ MSS mouse model.

Evaluation of the SIL1-deficient quadriceps proteome revealed that loss of SIL1 not only had a large effect on the expression of secretory pathway proteins that are synthesized in the ER, but also led to significant changes in all major cellular organelles, as well as pathways critical for normal muscle physiology. Our interpretation of the causes of these wide-ranging effects on cellular homeostasis is as follows (Fig. 7). The loss of SIL1, a NEF for the ER chaperone BiP, results in an imbalance in the protein-folding capacity of the ER, as evidenced by post-translational alterations in the levels of multiple secretory pathway clients and the presence of ER chaperones partially partitioning in the NP40-insoluble fraction, putatively bound to secretory pathway protein aggregates. This results in the upregulation of ER chaperones and components of multiple cellular protein degradation pathways due to an increased demand for both. However, these responses seemed unable to wholly compensate for an apparent perturbation in ER homeostasis. As a result, cellular protein degradation pathways are heavily taxed, secondarily affecting the turnover of proteins throughout the cell, as evidenced by the accumulation of ubiquitinated proteins, cytosolic sHSP chaperones fractionating in the detergent-insoluble fraction, likely bound to protein aggregates, and the presence of electron-dense material and cytosolic bodies detected by EM. Multiple studies document a basal activation of the UPR in normal adult murine skeletal muscles (Iwawaki et al., 2004; Acosta-Alvear et al., 2007; Muñoz et al., 2013), which contributes to myogenesis (Blais et al., 2005) and adaptation to exercise (Wu et al., 2011). Thus, the upregulation of numerous effectors of the UPR observed in our study indicates an increase that exceeds basal activation. However, the basal engagement of the UPR in adult skeletal muscles may restrict the extent to which this pathway can be further upregulated physiologically in the event of a disruption in ER homeostasis, as observed upon SIL1 loss. This would explain why, despite UPR activation, the SIL1-deficient ER has not been able to completely resolve its proteostasis defects.

In keeping with a disruption in ER homeostasis, we identified significant decreases in the expression of IR-β and IGF1R-β, which are synthesized in the ER and likely fail to mature properly under these conditions, leading to their disposal by ERAD or lysosomal systems. Although we observed decreases in the upstream transducers of this pathway, paradoxically we found evidence of higher basal stimulation of downstream components in *Sil1*^Gt^ quadriceps. It is noteworthy that mice with a muscle-specific dual knockout of IR and IGF1R also displayed a compensatory basal activation of the PI3K-AKT-mTOR pathway and increased surface expression of the GLUT1 and GLUT4 glucose transporters (O'Neill et al., 2015), confirming that skeletal muscles can respond to decreased (or completely absent) levels of these two receptor tyrosine kinases by constitutively inducing their shared effector pathway. Interestingly, spliced X-box-binding protein-1 (sXBP1) has been demonstrated to preserve sensitivity of the PI3K-AKT signaling axis during high-fat-diet-induced ER stress by negating the inhibitory effects of IRE1-mediated JNK activation (Ozcan et al., 2004). Thus, the concomitant upregulation of *sXbp1* in *Sil1*^Gt^ quadriceps is likely to contribute to the maintenance of a sensitive growth factor signaling axis in the face of decreased IR and IGF1R levels.

Skeletal muscle mass is maintained by a dynamic balance between anabolic (hypertrophic increase in protein synthesis rates) and catabolic (atrophic increase in protein degradation pathways) processes. Signaling through the PI3K-AKT pathway positively regulates muscle growth and mass by increasing cellular protein synthesis through mTOR signaling (Laplante and Sabatini, 2012; Glass, 2010) and AKT-dependent phosphorylation of FOXO transcription factors to suppress their atrophy-inducing activity. Although we observed both these cues in *Sil1*^Gt^ quadriceps, muscle mass continued to decline, suggesting that these counteracting mechanisms were insufficient to prevent atrophy. It is intriguing to hypothesize that the seemingly compensatory activation of mTOR signaling in *Sil1*^Gt^ skeletal muscles, which would maintain high protein synthesis rates and inhibit autophagy (Kim et al., 2011), might in fact further exacerbate the demands on an already abnormally taxed protein folding machinery. In support of this hypothesis, there is a growing body of literature that emphasizes the beneficiary effect of reduced PI3K-AKT-mTOR signaling on reducing proteotoxicity associated with protein misfolding diseases in worms and mice (Cohen et al., 2009; Morley et al., 2002). In keeping with this hypothesis, overexpression of the translation inhibitor 4E-BP dramatically suppressed pathological phenotypes in a *Drosophila* model of Parkinson's disease (Tain et al., 2009), and inhibition of mTOR with rapamycin abolished cognitive defects and reduced β-amyloid levels in a murine Alzheimer's disease model (Spilman et al., 2010).

Aging is the principal risk factor in various protein folding diseases, such as BAG3-, CRYαB- and DNAJB6-associated myofibrillar myopathies, as well as familial amyotrophic lateral sclerosis, Alzheimer's, Huntington's and Parkinson's diseases, all of which have been characterized as ‘gain-of-toxic-function’ diseases (Taylor et al., 2002). Although all of these diseases result from germline-encoded mutation(s), disease-associated symptoms and even aggregation of the affected proteins does not occur until later in life. This has been attributed to a progressive, age-related, decline in chaperone function, clearance of aberrantly folded proteins and the ability to mount successful stress responses, all of which collectively maintain the proteome integrity. These changes have been documented in multiple tissue types, including muscles, and in organisms ranging from nematodes to fruit flies and mammals (Taylor and Dillin, 2011). This decline in proteostasis maintenance during aging predicts that, as the buffering capacity of the chaperones and stress responses diminish, misfolded proteins begin to aggregate. Powers et al. (2009) have used mathematical modeling to argue that there are limits on the capacity of the proteostasis network in cells and that metastable proteins would be the first to be affected as this network declines. Although SIL1 is a component of the ER chaperone machinery and might be expected to affect the proper maturation of numerous secretory pathway proteins, we also observed alterations in protein expression in other subcellular organelles and a similar delay in the onset of physiological symptoms and molecular responses. Thus, we argue that the resulting disruption of ER function in *Sil1*^Gt^ skeletal muscles provides a sufficient burden to accelerate the usual age-restricted limitations of the proteostasis network. Furthermore, we find that the alterations in ER homeostasis secondarily impinge on proteostasis in other organelles, supporting the hypothesis that organellar boundaries are susceptible to disruption in *trans* (Powers et al., 2009).

One might speculate that any of the secretory pathway proteins that show altered expression upon *Sil1* disruption represent clients that are critically dependent on SIL1 for release from BiP. However, alterations in the expression of the secretory pathway proteins that we examined did not become apparent until the mice were 6 months of age. This argues that *Sil1*^Gt^ mice were able to control the expression of these proteins normally in the absence of SIL1 at earlier time points and raises the question of why they are unable to do so later. It is possible that basal levels of GRP170, another ER NEF for BiP, are initially sufficient to compensate for the loss of SIL1 function but cannot do so at later time points. Alternatively, an intriguing possibility comes from recent studies in yeast showing that Sil1p (yeast SIL1) can also act as a reductase for oxidized yeast BiP (Kar2p), thereby restoring Kar2p's chaperone activity during recovery from oxidative stress. Sil1p's reductase activity is mediated through two cysteines present near its N-terminus (Siegenthaler et al., 2017). Although there are two N-terminal cysteines that are highly conserved in mammalian SIL1 sequences, algorithms for signal sequence cleavage predict that they are not retained in the mature protein. If in fact they are part of the mature SIL1 protein, a similar reductase activity for mammalian SIL1 could readily explain the delayed effects on secretory pathway protein expression, as the need for this reductase activity might be dependent on accumulated oxidative insults to the ER, leading to the oxidization and inactivation of BiP. Further investigation is required to determine if a mammalian SIL1 possesses a reductase activity for BiP and whether this is a plausible explanation for the pathologies associated with MSS.

*In toto*, our study revealed that loss of SIL1 leads to early and persistent defects in ER homeostasis, which invokes a triage response to increase both the protein folding and degradation capacity of the ER, which is ultimately inadequate. One consequence of the inability to restore proper ER function is a post-translational decrease in IR-β and IGF1R-β expression in SIL1-deficient skeletal muscles. While we observed evidence of a compensatory increase in basal PI3K-AKT-mTOR signaling and expression of glucose transporters in the *Sil1*^Gt^ skeletal muscles, these mice were not able to successfully counteract the activation of atrophy-causing degradation pathways. Thus, the physiological requirement of constitutive UPR activation in skeletal muscles, combined with the proteotoxicity associated with increased mTOR signaling and the age-associated decline in the ability to maintain proteostasis, particularly in the absence of a pronounced regenerative response, is likely to trigger the loss of skeletal muscle mass and strength associated with SIL1 loss. Together, these findings provide molecular insights into the progressive myopathy and cellular triage responses attempted upon loss of a component of the ERQC machinery.

## MATERIALS AND METHODS

### Mice

The *Sil1*^Gt^ mouse strain B6;129Sil1^Gt(pGT2TMpfa)1Slac^ was generated by gene-trap methodology (Zhao et al., 2005) and was obtained from Dr Susan Ackerman (HHMI and University of California, San Diego, CA). Heterozygous mice were crossed to generate homozygous male and female *Sil1*^Gt^ mice and wild-type littermates. At weaning, DNA was isolated from mouse tail snips using DNeasy^®^ Blood & Tissue Kit (Qiagen, Valencia, CA) and genotyped as previously reported (Zhao et al., 2005). The primers for the wild-type allele and for the disrupted allele are mentioned in Table S1. Mutant mice were born at expected Mendelian ratios. Mice were housed and treated in accordance with the Animal Use and Care Committee and the Animal Research Center at St. Jude Children's Research Hospital, adhering to the National Institutes of Health guidelines.

### Inverted screen test

To measure the muscular strength of male and female *Sil1*^Gt^ and wild-type mice, we performed an inverted screen test (Deacon, 2013) using a 12″×8″ screen (0.5″ metal grid) that was surrounded by 9″ plexiglass walls to prevent mice from climbing onto the top of the screen during the assay. A pillow was placed below the apparatus to cushion the fall. Each mouse was placed on the screen, allowed to acclimatize for a few seconds, and the screen was rotated 180° to position the mouse upside down, at which point timing began. The length of time that each mouse was able to hold on to the screen was recorded with an assay maximum limit of 120 s. Each mouse was subjected to a total of three trials successively and the average was determined. Mean times for wild-type and *Sil1*^Gt^ mice of each age group were statistically compared using Prism Ver. 6 (GraphPad Software, La Jolla, CA) by Mann–Whitney tests (unpaired; two-tailed).

### Histopathology and immunohistochemistry

Immediately after euthanization, mice were perfused with 10% buffered formalin (ThermoFisher Scientific, Waltham, MA) through the left cardiac ventricle. Skeletal muscles and spinal cords of perfused mice were post-fixed by immersion in 10% buffered formalin for at least 24 h before being decalcified in formic acid (TBD-2 Decalcifier; Thermo Fisher Scientific). Tissues were embedded in paraffin, sectioned at 4 μm and mounted on positively charged glass slides (Superfrost Plus; Thermo Fisher Scientific). Tissue sections were processed for IHC using the automated Discovery Ultra platform (Ventana-Roche, Tucson, AZ) after heat-induced antigen retrieval for up to 48 min using the Discovery Cell Conditioning CC1 buffer, which included proteinase K treatment in the case of laminin IHC. Sections were then stained with the respective primary and secondary antibodies, counter-stained with Hematoxylin II followed by treatment with Bluing Reagent, and detected using the DISCOVERY ChromoMap DAB Kit (RUO) alone or in conjunction with the DISCOVERY Purple Kit (RUO) for the double-staining of MyHC fast and slow isoforms. Reagents used are listed in Table S1.

### Transmission electron microscopy

For ultrastructural analysis by EM, post-euthanization the animals were perfused with 2.5% glutaraldehyde/2% paraformaldehyde in 0.1 M sodium cacodylate buffer, pH 7.4 (EM fixative). Necropsy was performed to harvest major skeletal muscles and heart, and submitted to the St. Jude CTI-EM shared resource. The samples were fixed with EM fixative overnight and post-fixed in 2% osmium tetroxide in 0.1 M cacodylate buffer with 0.3% potassium ferrocyanide for 1.5 h. After rinsing in the same buffer, the tissue was stained in 4% aqueous uranyl acetate and dehydrated through a series of graded ethanol-to-propylene oxide transitions. The samples were then infiltrated in a series of propylene oxide and EPON resin gradients, with processing through 100% EPON resin overnight. All processing was performed using a Leica EM TP Tissue Processor. The following day, tissues were embedded using fresh EPON resin and polymerized at 70°C overnight. Semi-thin sections (0.5 μm) were stained with Toluidine Blue for light-microscopy examination. Ultrathin sections (80 nm) were cut and imaged using the Jeol 1200 Electron Microscope with a 2K AMT 2K Digital Camera.

### Mass spectrometry

#### Protein digestion and peptide isobaric labeling by Tandem Mass Tags (TMTs)

Mass spectrometry was performed as previously described (Bai et al., 2017) with a slight modification. Quadriceps tissues from five *Sil1*^WT^ and five *Sil1*^Gt^ 6-month-old male mice were lysed in a 50 mM HEPES (pH 8.5)-based buffer containing 8 M urea and 0.5% sodium deoxycholate. Protein concentration of lysates was determined by a Coomassie-stained short gel with bovine serum albumin as standard. 100 µg of protein for each sample was digested with LysC (Wako) at an enzyme-to-substrate ratio of 1:100 (w/w) for 2 h at room temperature. Following this, the samples were diluted to a final concentration of 2 M urea in 50 mM HEPES (pH 8.5) and further digested with trypsin at an enzyme-to-substrate ratio of 1:50 (w/w) for at least 3 h. The digestion was terminated and acidified by adding trifluoroacetic acid to 1%, desalted using C18 cartridges and dried by SpeedVac. The purified peptides were resuspended in 50 mM HEPES (pH 8.5), labeled with 10-plex TMT reagents, following the manufacturer's recommendation (ThermoFisher Scientific).

#### Two-dimensional HPLC and mass spectrometry

The TMT-labeled samples were mixed in an equal proportion, desalted and fractionated on an offline high-performance liquid chromatography (HPLC) apparatus (Agilent 1220) using basic pH reverse-phase liquid chromatography (pH 8.0, XBridge C18 column, 4.6 mm×25 cm, 3.5 μm particle size). The fractions were dried and resuspended in 5% formic acid and analyzed by acidic pH reverse phase LC-MS/MS analysis. The peptide samples were loaded on a nanoscale capillary reverse phase C18 column (New Objective, 75 μm ID×∼40 cm, 1.9 μm C18 resin from Dr Maisch, GmbH) by an HPLC system (Waters nanoAcquity) and eluted by a 150 min gradient. The eluted peptides were ionized by electrospray ionization and detected by an inline Orbitrap Fusion mass spectrometer (ThermoFisher Scientific). The mass spectrometer is operated in data-dependent mode with a survey scan in Orbitrap (60,000 resolution, 2×106 AGC target and 50 ms maximal ion time) and MS/MS high-resolution scans (60,000 resolution, 1×105 AGC target, ∼150 ms maximal ion time, 38 HCD normalized collision energy, 1 m/z isolation window and 20 s dynamic exclusion).

#### Mass spectrometry data analysis and bioinformatics

The MS/MS raw files were processed by a newly developed tag-based hybrid search engine, JUMP, which displayed better sensitivity and specificity than commercial package (e.g. Proteome Discoverer) (Wang et al., 2014). The data was searched against the UniProt mouse database concatenated with a reversed decoy database for evaluating false discovery rate. Searches were performed using a 25 ppm mass tolerance for precursor ions and 15 ppm mass tolerance for fragment ions, fully tryptic restriction with two maximal missed cleavages, three maximal modification sites, and the assignment of *a*, *b* and *y* ions. TMT tags on lysine residues and N-termini (+229.162932 Da) were used for static modifications, and Met oxidation (+15.99492 Da) was considered as a dynamic modification. MS/MS spectra were filtered by mass accuracy and matching scores to reduce protein false discovery rate to ∼1%. Proteins were quantified by summing reporter ion counts across all peptide spectrum matches using the JUMP software suite.

Bioinformatics analyses were performed to determine significant protein expression, gene ontology (GO) and pathway representation. Using Partek Genomics Suite 6.6 (Partek Incorporated, St Louis, MO), the values of 5384 quantified proteins were log_2_-transformed, followed by quantile normalization, and compared using ANOVA between five replicates in each of the two groups. Expressions of a total of 515 proteins were identified as significantly altered (*P*<0.01; log_2_ ratio <−0.5 or >0.5) in the *Sil1*^Gt^ quadriceps relative to those from age-matched wild-type mice. DAVID (https://david.ncifcrf.gov/) Bioinformatics Resources 6.7 was then used for GO analysis. Annotations for cellular components were available only for 490 proteins out of 515, which were then manually curated using the GeneCards database (www.genecards.org) to compute the percentage of proteins belonging to major cellular compartments according to their score. Gene-set enrichment analysis (http://software.broadinstitute.org/gsea/) was further performed to identify significant enrichments in differentially expressed gene signatures, including pathways based on gene-sets defined from KEGG, BioCarta and Reactome pathway databases.

### Western blotting

Male *Sil1*^Gt^ and wild-type mice (*n*=3 for each genotype and age group, with the exception of the 6-month time point, for which five male mice of each genotype were used) were used for western blotting studies. Immediately after euthanization, mice were perfused with ice-cold PBS through the left cardiac ventricle. Quadriceps were harvested at necropsy and flash frozen in liquid nitrogen, and either processed immediately or stored until further use. The frozen tissue was pulverized using a liquid-nitrogen-chilled mortar and pestle, and stored at −80°C until further use. The pulverized tissue was lysed, as described for mass spectrometry analysis, normalized for equivalent protein concentration using a BCA Protein Assay Kit (Pierce–ThermoScientific, Rockford, IL) and samples from the indicated number of mice at the same age were pooled. To separate NP40-soluble proteins from -insoluble ones, pulverized tissue was lysed in 50 mM Tris (pH 7.5) buffer containing 150 mM NaCl, 0.5% Nonidet P-40 and 0.5% deoxycholic acid. The clarified lysates obtained after centrifugation at 21,130 ***g*** for 20 min were designated as the NP40-soluble fraction. The NP40-insoluble pellets were washed once with NP40 lysis buffer and then solubilized in 50 mM Tris (pH 8) buffer, containing 0.6% SDS, sonicated for 4 min (30 s ON-30 s OFF cycles) and incubated at 95°C for 10 min. All buffers contained 0.25 mM phenylmethylsulphonyl fluoride (PMSF) and a protease inhibitor cocktail (Complete, Roche). In all cases, tissue lysates were electrophoresed under reducing conditions on gels ranging from 8% to 16%, or on 4% to 20% gradient gels (Mini-PROTEAN^®^ TGX™ Precast Protein Gels, Bio-Rad, Inc., Hercules, CA), depending on the size of the examined protein. Antibodies used for immunoblotting are listed in Table S1. HSC70 and GAPDH served as loading controls. Western blots from the pooled samples in each of the figures were quantified using either Image Studio™ Lite (LI-COR Biosciences, Inc., Lincoln, NE) or ImageQuant™ TL (GE Healthcare Life Sciences, Inc., Issaquah, WA). These values were normalized to their respective GAPDH control values and graphically expressed as fold change in *Sil1*^Gt^ protein expression, relative to the wild-type levels set to 1, at each age. Statistical analyses (Figs S4F and S5J) were performed using Prism Ver. 6 (GraphPad Software) by Student's *t*-tests (unpaired; two-tailed).

### Real-time PCR

Three male *Sil1*^Gt^ and wild-type mice for each genotype and age-group, respectively, were used for quantitative PCR (qPCR) studies. Total RNA was isolated from pulverized skeletal muscle using RNeasy^®^ Fibrous Tissue Mini Kit (Qiagen), according to the company protocol. 1 μg total RNA was reverse transcribed using the High Capacity cDNA Reverse Transcription Kit (Applied Biosystems) and subjected to SYBR^®^ Green chemistry-based qPCR using the QuantStudio 3 Real-time PCR System (Applied Biosystems, Foster City, CA). Primers were obtained from ThermoFisher Scientific. *Gapdh*, *Hprt1*, *Rer1*, *Rn18s* and *Rpl41* were tested as potential endogenous reference controls, as indicated (Thomas et al., 2014). *Gapdh*, *Rer1* and *Rpl41* were comparable in expression between the wild-type and the *Sil1*^Gt^ quadriceps and, thus, used as controls. Primer sequences are listed in Table S1. Relative mRNA fold change was computed from the QuantStudio-generated *C*_t_ values by using the 2^−ΔΔCt^ method. Wild-type mRNA levels were normalized to 1, and *Sil1*^Gt^ mRNA levels were plotted as a relative measure of the wild-type levels. Statistical analyses were performed using Prism Ver. 6 (GraphPad Software) by Student's *t*-test (unpaired; two-tailed).

### Skeletal muscle glucose uptake

Small animal PET-μCT was performed on docked Siemens Inveon DPET and micro-CT (Siemens Medical Solutions USA, Inc., Malvern, PA) instruments. Four 6-month-old wild-type and *Sil1*^Gt^ male mice were fasted overnight, anesthetized with 2% isoflurane, and placed supine on a heated animal bed, following which an intraperitoneal catheter was placed for ^18^F-FDG injection. Whole-body dynamic PET scan was acquired over 60 min. ^18^F-FDG (100-120 μCi, 200 μl) was injected via the i.p. catheter 1 min after initiating scanning. μCT data were acquired using a 2048×3072 mm Field of View with whole body coverage achieved using automatic stitching of two bed positions (36% overlap). Projections were acquired at 80 kVp and 500 μA (250 ms exposure) over a rotation of 180° (120 steps), providing an isotropic resolution of 107 μm, and used for PET attenuation correction. PET images were reconstructed by three-dimensional Ordered Subset Expectation Maximization (OSEM 3D) with binning scheme of 5-20, 10-60, 5-180, 5-240 and 2-400 (number of bins-time in seconds). The Inveon Research Workspace (IRW) software was utilized for data processing and image co-registration. Volumes of interests (VOIs) were drawn over the gastrocnemii. Data from these VOIs were used to calculate radioactivity accumulation for each animal and plotted versus time. Statistical comparisons of %ID/g were performed by Student's *t*-tests (unpaired; two-tailed) using Prism Ver. 6 (GraphPad Software).

## Supplementary Material

Supplementary information
