# Supplementary material for: SIL1, the endoplasmic-reticulum-localized BiP co-chaperone, plays a crucial role in maintaining skeletal muscle proteostasis and physiology
Source: Dis Model Mech. 2018 May 10;11(5):dmm033043. doi: 10.1242/dmm.033043 (PMC5992605; doi:10.1242/dmm.033043)
Supplement: Supplementary information [file dmm-11-033043-s1.pdf]

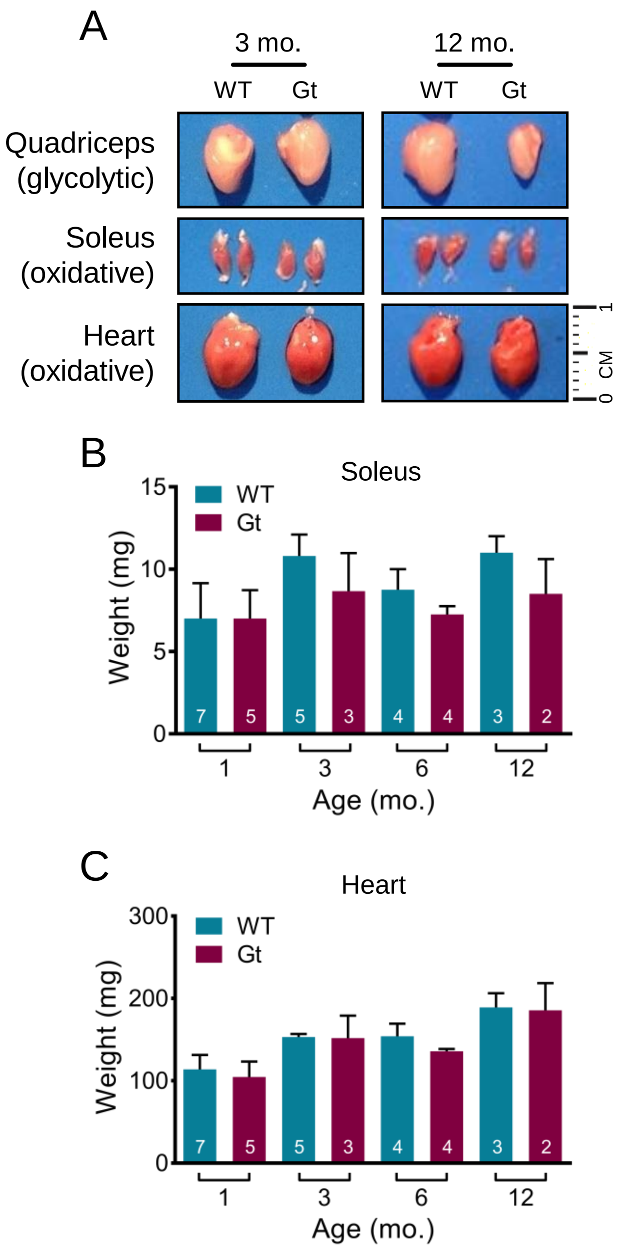

Fig. S1. Loss of SIL1 predominantly affects glycolytic skeletal muscles. (A) Representative images of quadriceps, soleus, and heart derived from wild-type and *Sil1*^{Gt} mice at the indicated ages depicting that loss of SIL1 specifically affects the glycolytic quadriceps, while sparing the oxidative skeletal muscle, soleus, and specialized cardiac muscle. **(B, C)** Comparison of wild-type and *Sil1*^{Gt} soleus (B) and heart (C) weights at the indicated ages. Error bars indicate means ± s.d. The number of mice in each group is indicated within the bars. Although there was a trend of slight decrease in the *Sil1*^{Gt} soleus weights, these values were not statistically different from the wild-type soleus weights.

Disease Models & Mechanisms • Supplementary information

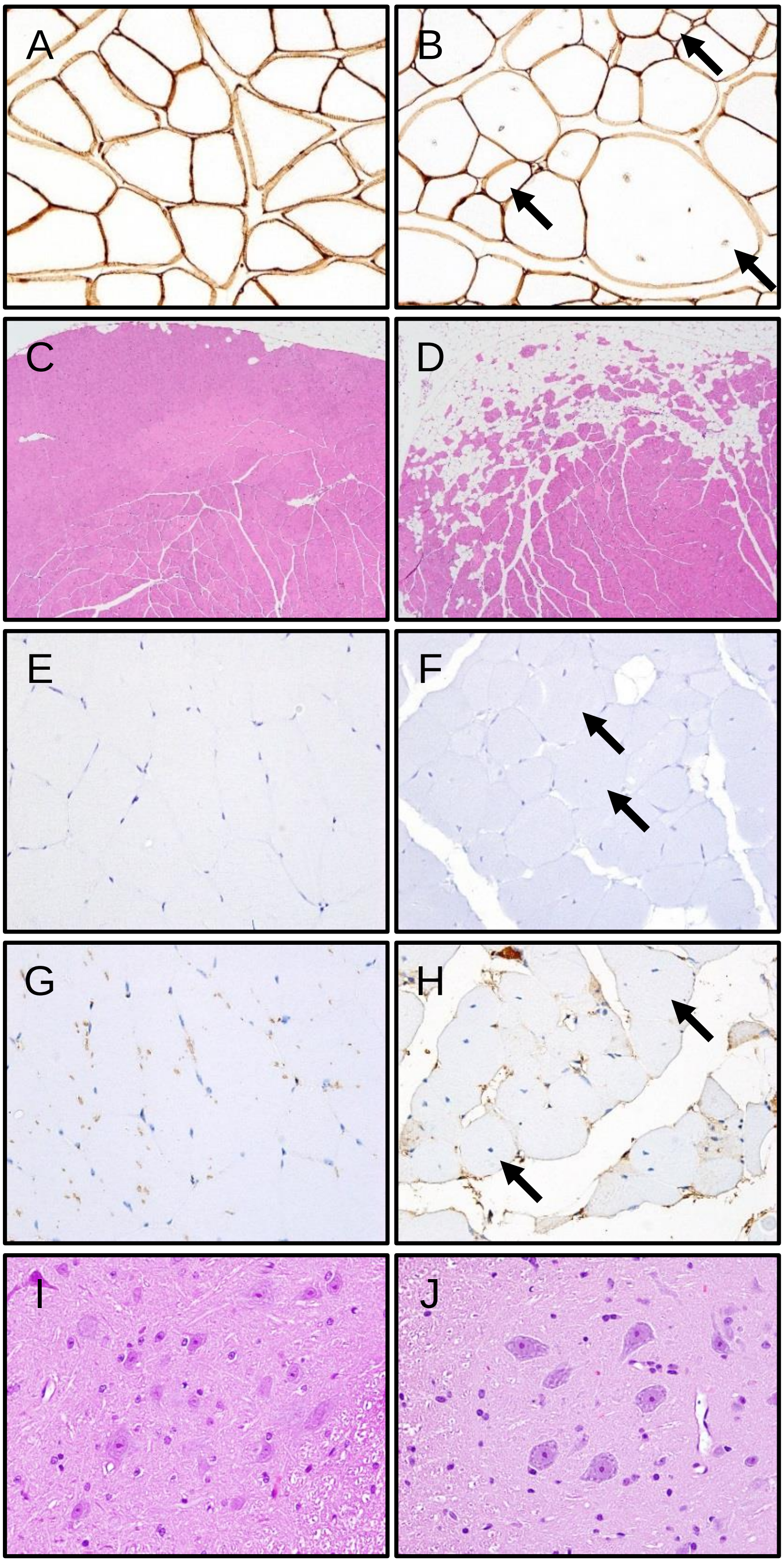

Fig. S2. ***Sil1*$^{\mathbf{Gt}}$ skeletal muscles display characteristic myopathic signatures.** **(A-J)** Representative hind-limb skeletal muscle cross-sections derived from 12-month-old wild-type (A,C,E,G,I) and *Sil1*^{Gt} (B,D,F,H,J) mice are stained with anti-laminin (A,B), an extracellular matrix protein; H&E (C,D) depicting adipose infiltration in peripheral skeletal muscles, predominantly affecting muscles in close proximity of adipose tissue; anti-immunoglobulins IgM (E,F) and IgG (G,H) to asses sarcolemmal integrity. (I,J) H&E staining of lumbo-sacral spinal cord sections display apparently normal motor neurons. Images were either captured at the same 40x (A-B,E-J) or 4x (C,D) magnifications, respectively.

Disease Models & Mechanisms • Supplementary information

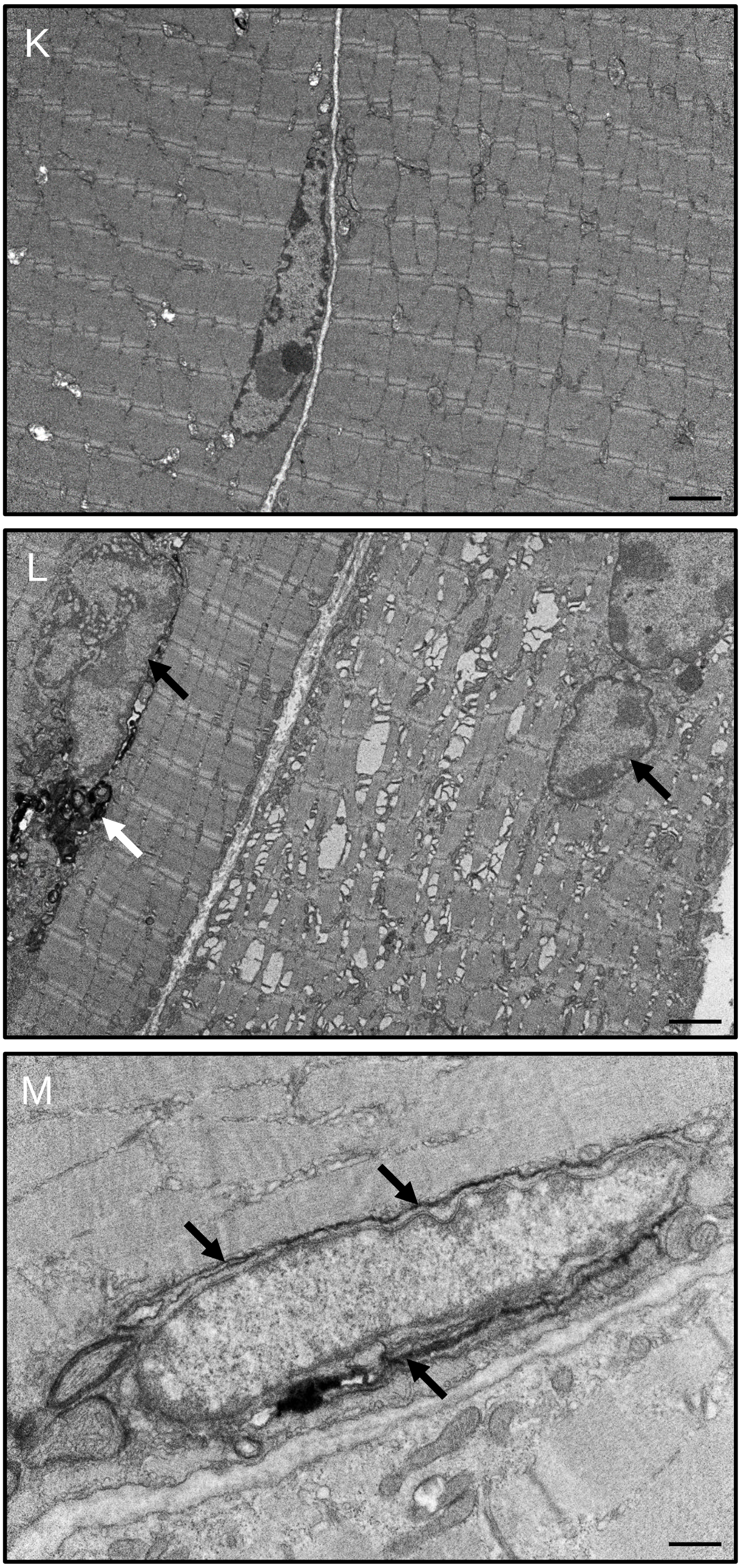

Fig. S2. ***Sil1***^{Gt} **skeletal muscles display characteristic myopathic signatures.** **(K-M)** Representative electron micrographs from 10-month-old wild-type (K) and *Sil1*^{Gt} (L,M) biceps brachii (K,L) and quadriceps (M) reveal multiple myopathic features, including degenerating myonuclei (L; black arrows), electron-dense aggregates (L; white arrow), disruption of the cytoskeletal architecture, and a double membranous structure surrounding the nucleus (M; arrows), which is characteristic of MSS, and implicated to be a nuclear envelope pathology in woozy mice (Roos et al., 2014). Scale bars: 2 µm (K,L); and 500 nm (M).

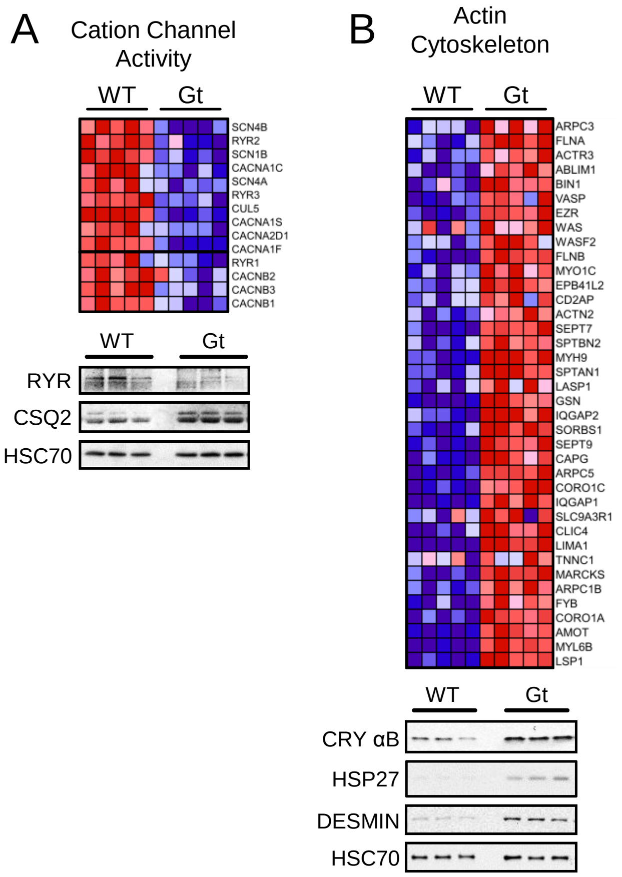

Fig. S3. Proteomic study reveals that loss of SIL1 affects pathways critical for normal muscle physiology. (A, B) Heat maps of (A) proteins involved in cation channel activity that were significantly depleted, and (B) proteins annotated to play a role in the actin cytoskeleton organization that were significantly enriched in *Sil1*^{Gt} quadriceps, respectively. A subset of proteins for each gene-set was selected for verification by western blotting and is shown below the heat maps. (A) Concomitant with a decrease in levels of the RYR Ca^{2+}-release channel subunits, proteomics indicated a significant increase in the levels of calsequestrin 2/CSQ2, which serves as the sarcoplasmic reticular Ca^{2+} buffering protein.

Disease Models & Mechanisms • Supplementary information

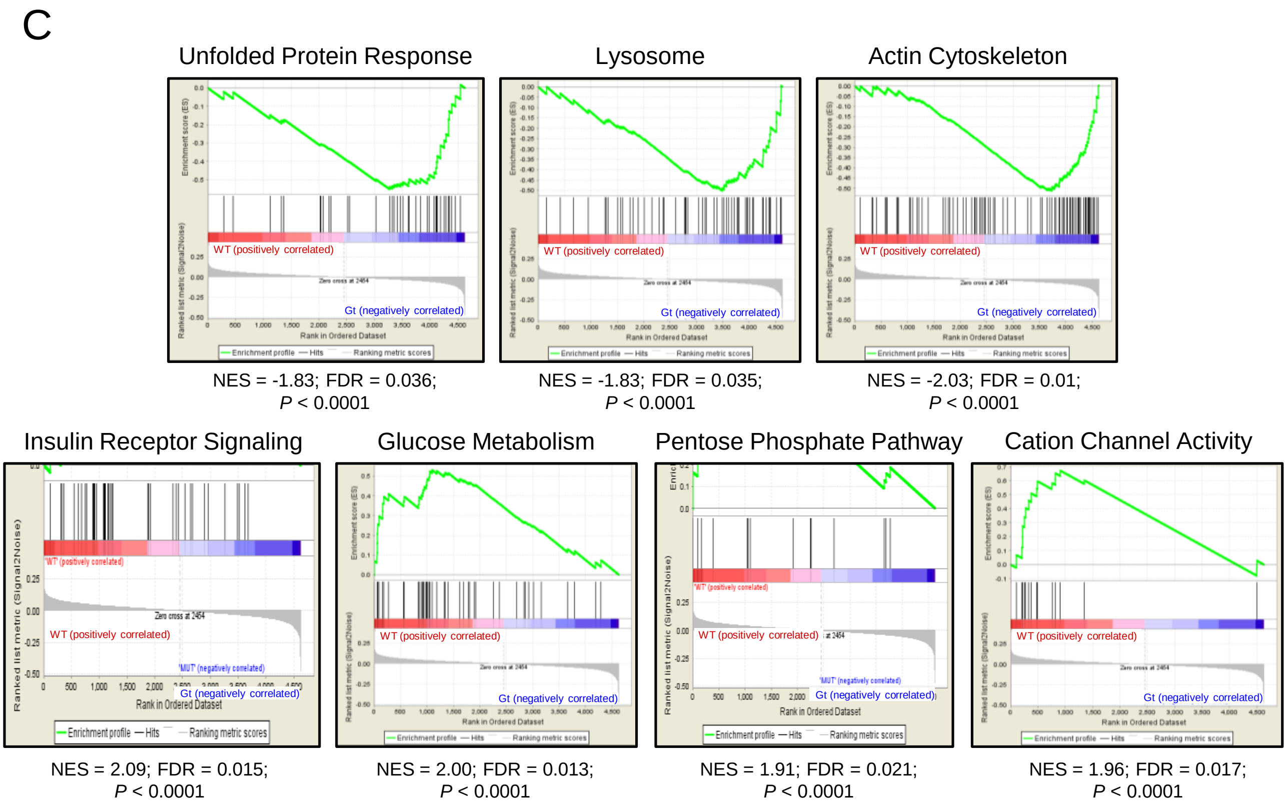

Fig. S3. Proteomic study reveals that loss of SIL1 affects pathways critical for normal muscle physiology. (C) GSEA-derived enrichment plots for the respective pathways and associated heat maps depicted in Fig. 3 and Fig. S3A-B. NES, normalized enrichment score; FDR, false discovery rate.

Disease Models & Mechanisms • Supplementary information

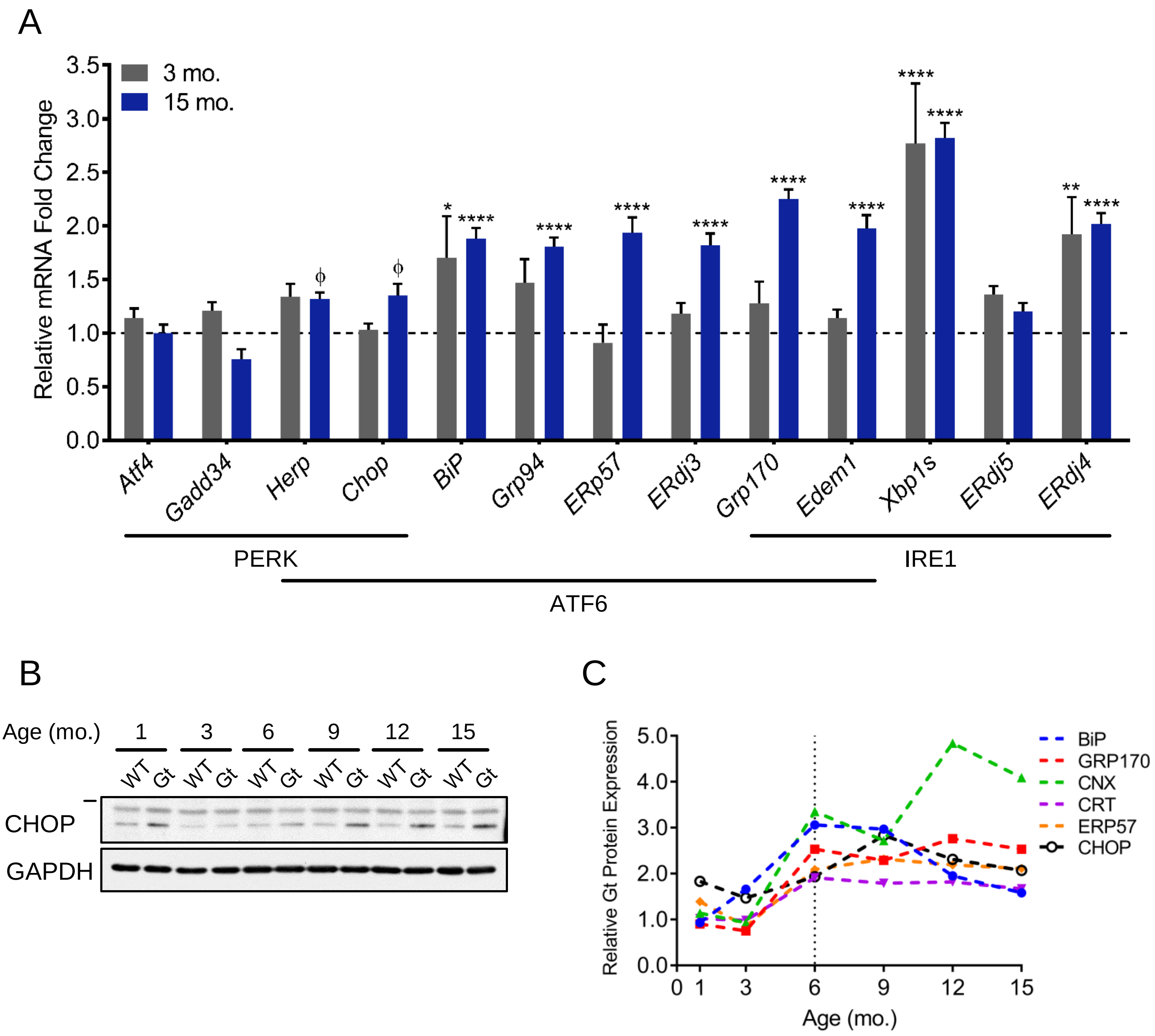

Fig. S4. Loss of SIL1 leads to activation of the Unfolded Protein Response and disruption of ER proteostasis. (A) Graphical representation of the indicated transcripts from *Sil1*^{Gt} quadriceps relative to the levels in wild-type quadriceps (*n*=3), and represented as fold change at 3 (gray) and 15 (blue) months. The arm of the UPR that regulates these components during ER stress is indicated below. Error bars indicate means ± s.e.m. Statistical differences were computed using unpaired, two-tailed Student's *t*-tests, and are indicated as $^{\phi}P$ <0.1 but >0.05, **P* ≤0.05, ***P* ≤0.01, ****P* ≤0.001, and *****P* ≤0.0001. **(B)** Urea-solubilized lysates from Fig. 4A were probed for CHOP. **(C)** Graph represent densitometric quantitation of western blotting data from Fig. 4 and Fig. S4B. Dotted vertical line at the 6-month time point indicates the age of onset of muscular weakness in *Sil1*^{Gt} mice, and at which time point quantitative proteomic profiling of quadriceps was carried out.

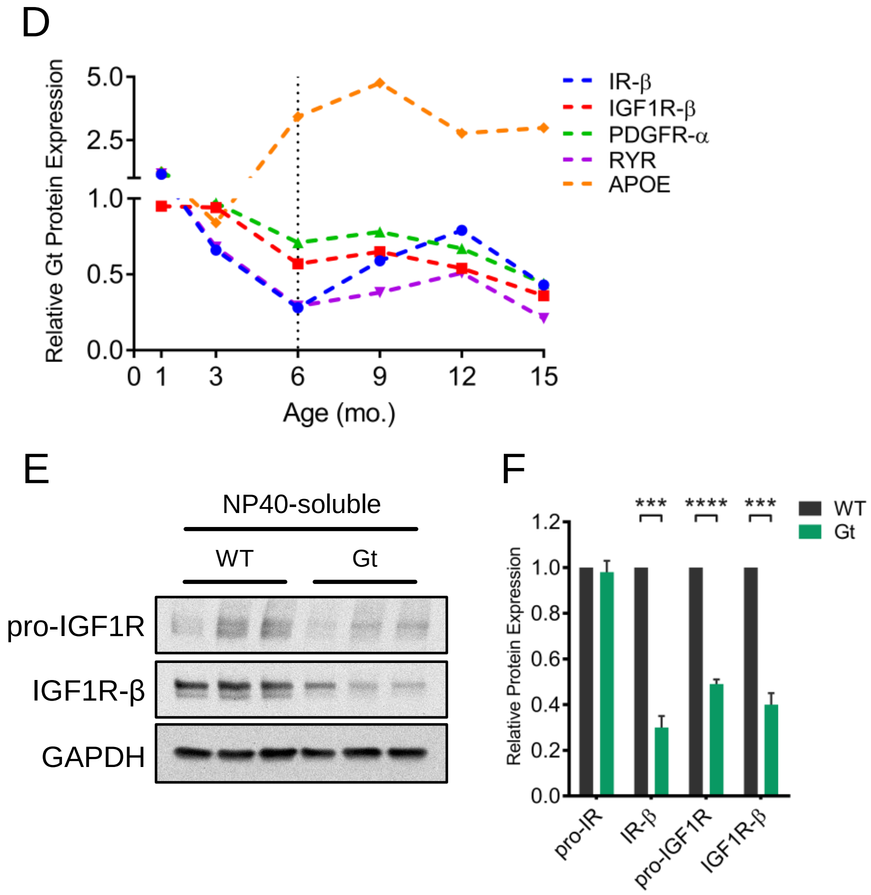

Fig. S4. Loss of SIL1 leads to activation of the Unfolded Protein Response and disruption of ER proteostasis. (D) Graphs represent densitometric quantitation of western blotting data from Fig. 4C. Dotted vertical line at the 6-month time point indicates the age of onset of muscular weakness in *Sil1*^{Gt} mice, and at which time point quantitative proteomic profiling of quadriceps was carried out. **(E)** NP40-solubilized quadriceps lysates from 12-month-old mice were processed for western blotting to detect the IGF-1 receptor precursor (pro-IGF1R) and the mature subunit (IGF1R-β). **(F)** Graph represent densitometric quantitation of western blotting data from Fig. 4D and Fig. S4E (n=3). Error bars indicate means ± s.e.m. Statistical differences were computed using unpaired, two-tailed Student's t-tests, and are indicated as ***$P \leq 0.001$ and **** $P \leq 0.0001$.

Disease Models & Mechanisms • Supplementary information

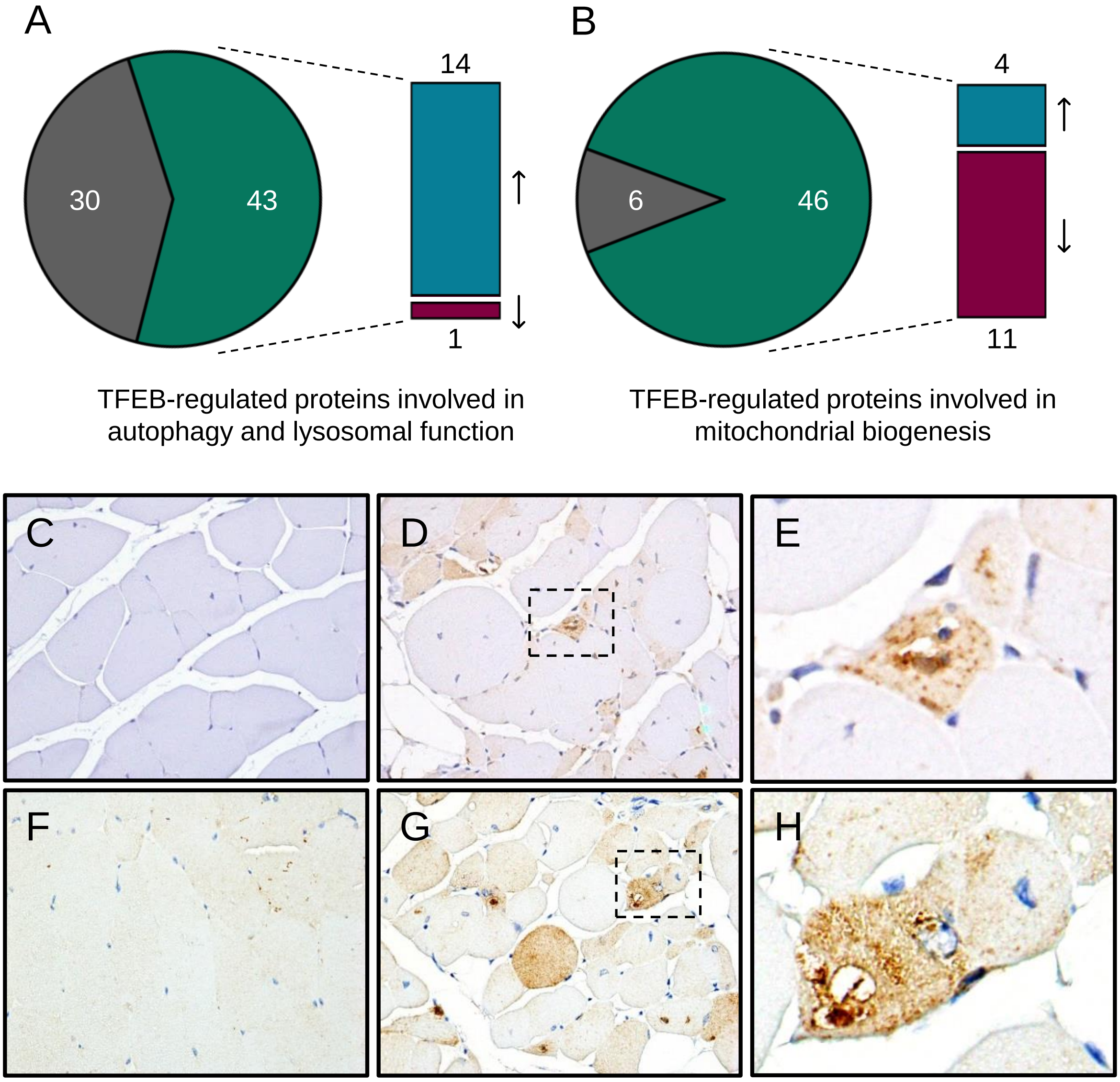

Fig. S5. Loss of SIL1 leads to aggregation of cytosolic small heat shock chaperones and disruption of cytosolic proteostasis. (A,B) Graphical representation of TFEB-regulated genes that were implicated in (A) autophagy and lysosomal function (Palmieri et al., 2011) or in (B) mitochondrial biogenesis (Mansueto et al., 2017). The total number of genes annotated in each study is represented by green and gray segments of each pie chart. The green segment of these pie charts (Table S3) represents the fraction of these TFEB-regulated genes whose proteomic signature was detected in our proteomics study. The bar chart adjacent to it displays the number of proteins significantly (log_2 ratio <-0.25 or >0.25; $P < 0.01$) upregulated (blue) or downregulated (red) in *Sil1*^{Gt} quadriceps, compared to wild-type muscles. **(C-H)** Representative hind-limb skeletal muscle cross-sections derived from 12-month-old wild-type (C,F) and *Sil1*^{Gt} (D,E,G,H) mice are stained with markers of autophagy, LC3B (C-E) and p62/SQSTM1 (F-H). (C,D,F,G) Images were captured at the same 40x magnification. (E,H) Display a higher magnification of the indicated area from (D,G), respectively.

Disease Models & Mechanisms • Supplementary information

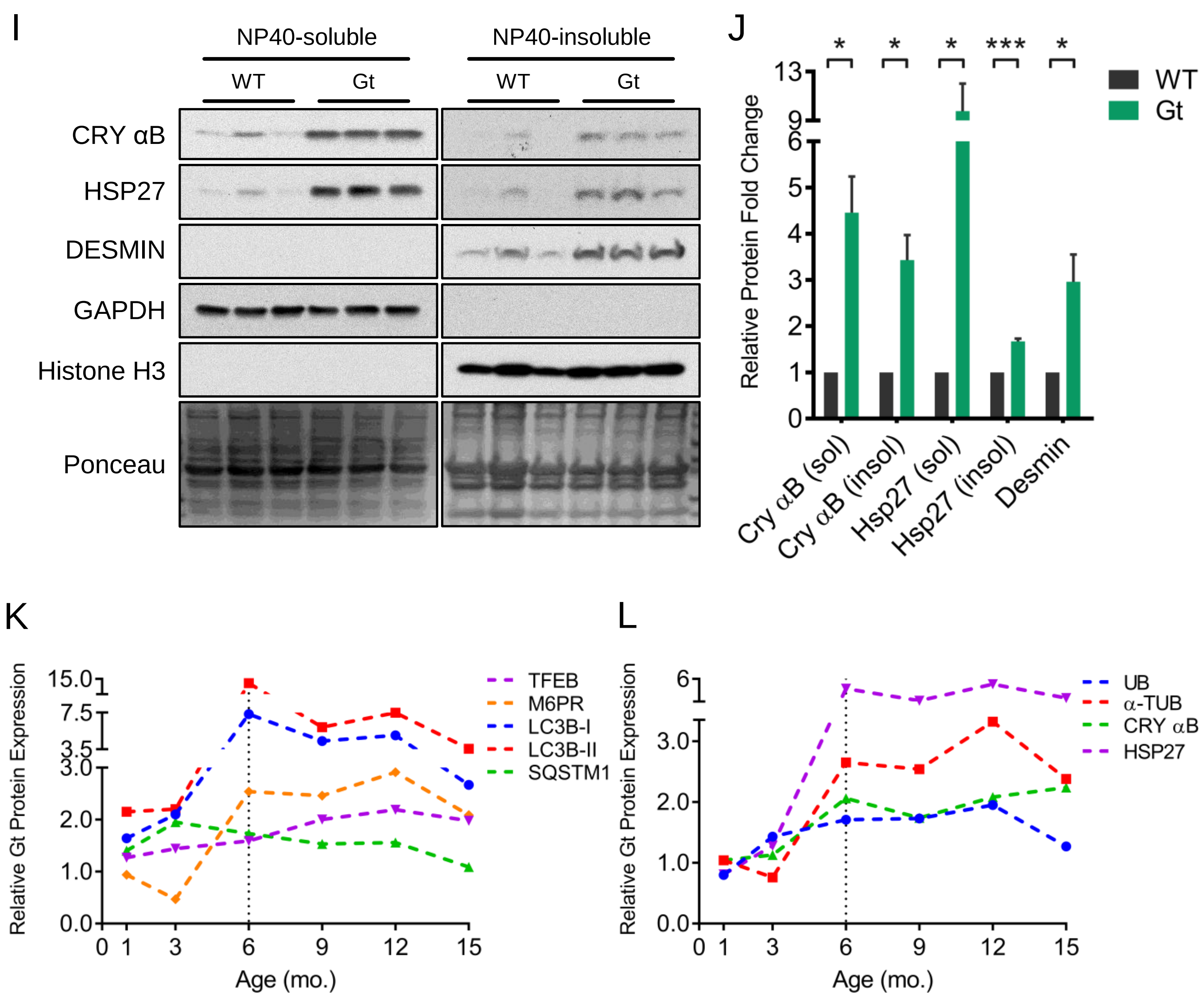

Fig. S5. Loss of SIL1 leads to aggregation of cytosolic small heat shock chaperones and disruption of cytosolic proteostasis. (I) Wild-type and *Sil1*^{Gt} quadriceps obtained from 12-month-old mice were lysed with NP40 lysis buffer. The resulting NP40-soluble and NP40-insoluble fractions were processed for western blotting for the indicated proteins. GAPDH and histone H3 serve as markers of NP40-mediated lysis efficiency. The Ponceau stained western blot serves as the loading control. **(J-L)** Graphs represent densitometric quantitation of western blotting data from Fig. S5I (*n*=3) and Fig. 5B-D. Error bars indicate means ± s.e.m. Statistical differences were computed using unpaired, two-tailed Student's *t*-tests, and are indicated as *$P \leq 0.05$ and *** $P \leq 0.001$.

Disease Models & Mechanisms • Supplementary information

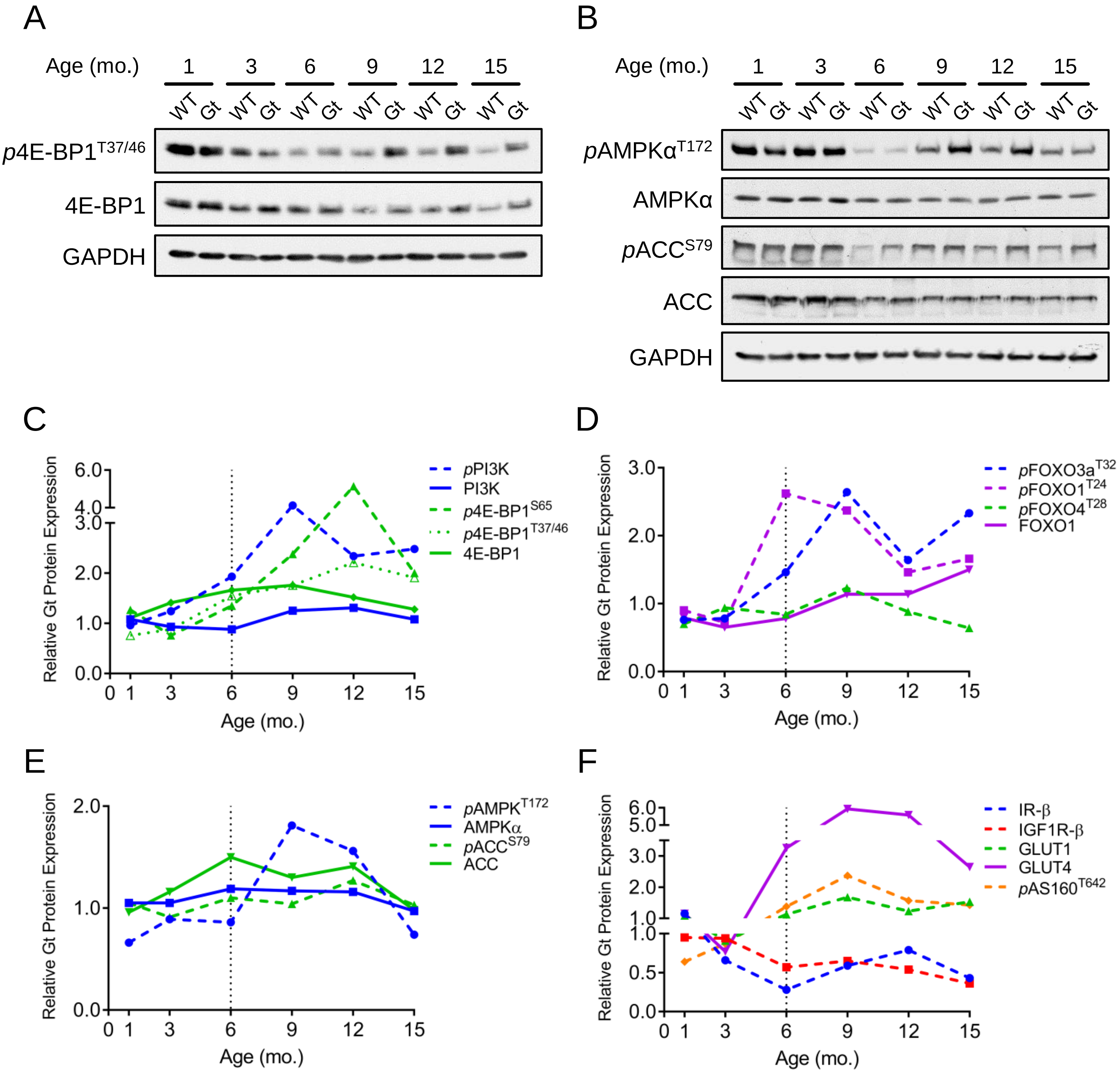

Fig. S6. Loss of SIL1 leads to basal activation of growth factor signaling. (A, B) Urea-solubilized quadriceps lysates derived from mice of the indicated genotypes and ages were probed with antibodies against phosphorylated and total 4E-BP1 (A), and phosphorylated and total levels of AMPKα and ACC (B). **(C-F)** Graphs represent densitometric quantitation of western blotting data from Fig. 6B,C and Fig. S6A,B.

Disease Models & Mechanisms • Supplementary information

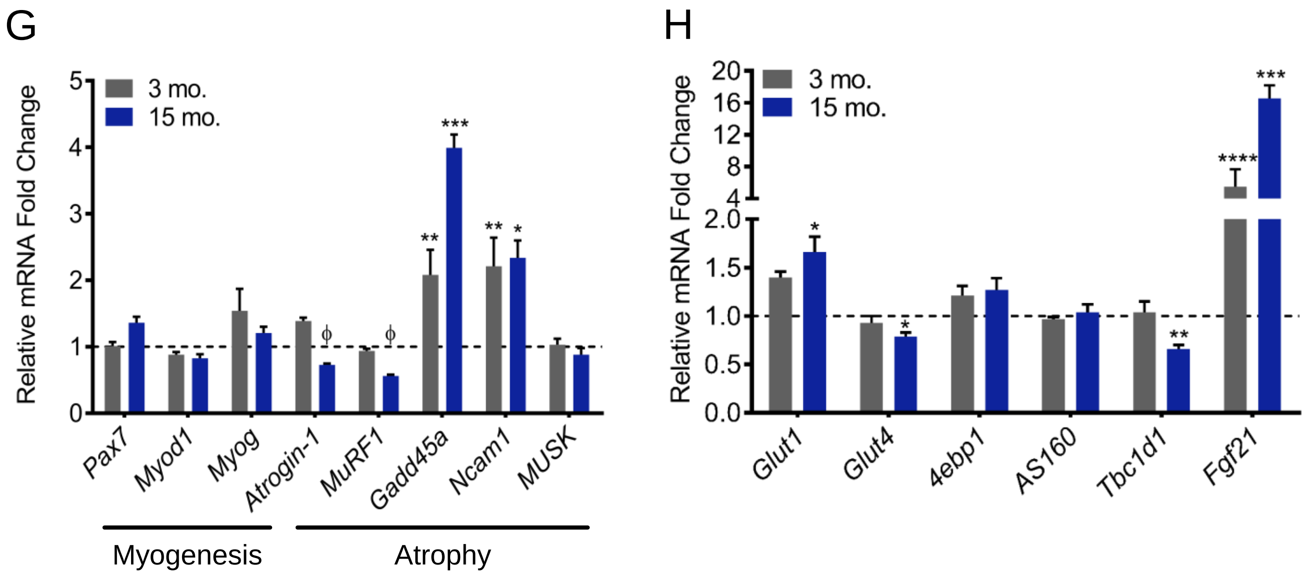

Fig. S6. Evaluating the expression of genes involved in skeletal muscle regeneration, atrophy, and glucose uptake. (G, H) Graphical representation of normalized mRNA fold changes in *Sil1*^{Gt} quadriceps (*n*=3) of genes involved in (G) satellite cell activation (*Pax7*), early myogenesis (*Myod1*), terminal myogenesis (*Myog*), atrophy (*Atrogin-1*, *MuRF1*, *Gadd45a, Ncam1*, *and MUSK*); or (B) glucose uptake (*Glut1*, *Glut4*, *AS160*/*Tbc1d4*, *Tbc1d1*, and *Fgf21*) at 3 (gray) and 15 (blue) months, respectively. Error bars indicate means ± s.e.m. Statistical differences were computed using unpaired, two-tailed Student's *t*-tests, and are indicated as $^{\phi}P$ <0.1 but >0.05, **P* ≤0.05, ***P* ≤0.01, ****P* ≤0.001, *****P* ≤0.0001.

Disease Models & Mechanisms • Supplementary information

Table S1. Key resources used, including information of antibodies used for western blotting and immunohistochemistry, primer sequences used for qPCR, and commercial assays, as well as identifying information of important reagents.

Click here to Download Table S1

Table S2. List of significantly altered proteins, detected by LC-MS/MS-based profiling of 6 mo.-old wild-type and $Sil1^{Gt}$ quadriceps.

Click here to Download Table S2

Table S3 A. List of significantly altered TFEB-regulated proteins that are involved in lysosomal function, detected by LC-MS/MS-based profiling of 6 mo.-old wild-type and $Sil1^{Gt}$ quadriceps. **B.** List of significantly altered TFEB-regulated proteins that are involved in mitochondrial biogenesis, detected by LC-MS/MS-based profiling of 6 mo.-old wild-type and $Sil1^{Gt}$ quadriceps.

Click here to Download Table S3
